# Unequal weathering: How immigrants’ health advantage vanishes over the life-course

**DOI:** 10.1016/j.jmh.2025.100303

**Published:** 2025-01-16

**Authors:** Silvia Loi, Peng Li, Mikko Myrskylä

**Affiliations:** a Max Planck Institute for Demographic Research, Rostock, Germany; b Max Planck–University of Helsinki Center for Social Inequalities in Population Health, Helsinki, Finland; c Helsinki Institute for Demography and Population Health, University of Helsinki, Helsinki, Finland

**Keywords:** Weathering hypothesis, Ageing, Health trajectories, Immigrant health

## Abstract

The weathering hypothesis implies that there is an interaction between age and race or ethnicity that results in marginalized groups experiencing a more rapid decline in health than the dominant groups. This hypothesis has been tested mostly focusing on racial and ethnic health inequalities, while less is known about weathering by immigration background. This paper aims at contributing to this strand of research by addressing four research questions: is the health of immigrants declining at a faster pace over the life-course, compared to non-immigrants? Do higher levels of education protect immigrants from accelerated ageing compared to non-immigrants? How do income and marital status affect the health trajectories of immigrants and non-immigrants? How do these patterns vary by sex? We use longitudinal survey data to estimate healthy ageing trajectories of immigrants and non-immigrants over the life-course, in the German context. We examine the roles of education, income, and marital status, separately for men and women. We find that immigrants, and especially immigrant women, have a faster health decline than non-immigrants; that high education is linked to higher levels of health, but does not protect immigrants from ageing in poorer health compared to non-immigrants; and that health disparities between immigrants and non-immigrants persist over the life-course net of the socio-economic controls, which appear to be secondary to other unobserved determinants.

## Highlights

• Immigrants experience faster health deterioration over time, leading to significant health disadvantages in older age.
• Lower education correlates with better initial health among younger immigrants, but this advantage diminishes with age, increasing disparities.
• Marriage and high income do not improve health outcomes for immigrants.
• Disparities in health between immigrant and non-immigrant women are more pronounced than among men, highlighting greater vulnerability.
• Immigrant women experience a shift to poorer health at an earlier age than immigrant men, indicating heightened risk.

## Introduction

Despite having better health upon arrival, the health of immigrants declines more rapidly than the health of non-immigrants as they age (Gubernskaya, 2014; Loi et al., 2024; Ronellenfitsch and Razum, 2004). The initial, paradoxical gap – known as the healthy immigrant effect (Abraído-Lanza et al., 1999) – is argued to be due to the health selectivity of individuals who successfully migrate to the receiving countries (Abraído-Lanza et al., 1999; Lechner and Mielck, 1998; Loi and Hale, 2019; Palloni and Arias, 2004; Ronellenfitsch and Razum, 2004). The health selection of immigrants is particularly strong upon arrival, and in the years immediately thereafter. However, this health advantage tends to diminish relatively quickly with the length of stay; around 10 years after arrival, most health differences between immigrants and non-immigrants vanish (Loi and Hale, 2019) – while immigrants age in the receiving societies (Kristiansen et al., 2016). Due to this loss of the health advantage, immigrants are at risk of accelerated ageing compared to non-immigrants.

Research indicates that compared to non-immigrants, immigrants live longer (Wallace et al., 2022), but in poorer health (Boen and Hummer, 2019; Wallace, 2024), living many years of their lives in relatively poor health. In fact, evidence shows that older immigrants have decreased physical functioning, higher rates of depression, and poorer self-rated health (Aichberger et al., 2010; Lanari and Bussini, 2012; Reus-Pons et al., 2018).

While recent studies adopt longitudinal approaches to examine immigrant health, further research is needed that extends our understanding of the dynamic of immigrant health over the life-course. In this work, we aim to contribute to this growing strand of research and explain how structural factors, both individually and in combination, contribute to and intensify health inequalities between immigrants and non-immigrants throughout the life-course.

The weathering hypothesis (Geronimus, 1992, 1996) suggests that the cumulative effects of social, economic, and environmental adversity accelerate health deterioration among marginalized populations, including immigrants. According to this hypothesis, chronic exposure to stressors, such as discrimination, economic hardship, and inadequate access to healthcare, leads to premature ageing and early onset of chronic diseases. The weathering hypothesis explains why health disparities persist even when controlling for socio-economic status, suggesting that the cumulative burden of lifelong stress erodes physiological resilience.

Drawing from the sociodemographic literature, and especially on the theoretical framework of the weathering hypothesis (Geronimus, 1992, 1996), we seek to understand whether immigrants age in poorer health compared to non-immigrants. We test whether education plays a protective role towards immigrants’ accelerated ageing. We study whether income and marital status contribute further to explaining health disparities between immigrants and non-immigrants over the life-course, and how these patterns vary by sex.

We demonstrate that immigrants experience a steeper decline in health with age, compared to non-immigrants, and widening health disparities over the life-course. We find that there is a limited protective role of education and of the socio-economic circumstances for immigrants. We show that immigrant women are particularly vulnerable, displaying steeper health trajectories and widening health disparities by migration background with age, compared to men.

## Theoretical background

Persistent exposure to socio-economic disadvantage and to lifelong stressors has been linked to more rapid health decline, and helps to explain ethnic and racial disparities on a range of health outcomes (Forde et al., 2019). In the literature, this process is often referred to as weathering (Geronimus, 1992, 1996). The process of weathering leads to accelerated ageing and to the early onset of chronic diseases and excess mortality among marginalized groups (Jones et al., 2019). As such, the process of weathering can be interpreted as a physical consequence of social inequality (Geronimus, 1996).

The weathering hypothesis was first introduced to study Black/White differences in the relationship between maternal age and birth weight and other perinatal health outcomes (Geronimus, 1992, 1996). Research that examined levels of neonatal mortality and of low birth weight among first births found that the age-specific distributions differ between Black and White women. In particular, it was shown that the health of Black women begins to deteriorate earlier in adulthood as a physical consequence of cumulative socio-economic disadvantages, whereas the health of White women starts to deteriorate at older ages (Geronimus, 1992; Wildsmith, 2002). Although research on the weathering hypothesis has primarily focused on racial and ethnic differences in perinatal health outcomes, this theoretical framework is well suited to help explain the wider adverse effects of cumulative disadvantage over the life course among any minority group, including immigrants, compared to the dominant groups (Wildsmith, 2002).

The evidence on weathering provides us with a fundamental insight: age is not only a biological developmental indicator, but it is also a reflection of the ways in which social inequality, discrimination, or bias in exposures to psychosocial or environmental hazards can lead to health differences between groups (Geronimus, 1992; Wildsmith, 2002). While there is a large body of literature on differential ageing by race and ethnicity, fewer studies have examined patterns of weathering by immigration background (Wildsmith, 2002), especially in the European context. The experience of marginalization, which immigrants often experience, can lead to chronic stress, which has been linked to worse health outcomes (Szaflarski and Bauldry, 2019). The stress caused by social exclusion, and systemic barriers faced by immigrants can result in a process of accelerated ageing and deteriorating health. This model can similarly describe how immigrants in Europe experience ongoing stressors that contribute to health disparities in comparison to non-immigrant populations.

There is evidence in the literature on migration and health that age does not relate to health in the same way among immigrants as it does among non-immigrants. Due to the healthy immigrant effect (Abraído-Lanza et al., 1999; Markides and Coreil, 1986; Moullan and Jusot, 2014), immigrants have better health than non-immigrants at younger ages. However, with increasing age, the health of immigrants deteriorates faster than the health of non-immigrants, which results in immigrants having poorer health than non-immigrants at older ages (Gubernskaya, 2014; Kristiansen et al., 2016; Jang et al., 2023; Loi et al., 2024).

Inequalities in health status appear to be related to exclusion and inequalities in socio-economic status (Kosteniuk and Dickinson, 2003; Lahelma et al., 2004; Mackenbach et al., 2015; Marmot et al., 2012). Common indicators of socio-economic status are education and income. Education impacts health indirectly by encouraging healthy behaviours, and directly by enabling access to medical care (Lahelma et al., 2004; Leigh, 1983; Mackenbach et al., 2015). Moreover, higher education provides individuals with a cultural supply that raises their awareness about their own health conditions and health prevention (Ross and Wu, 1995).

However, the distribution of education between immigrants and non-immigrants is unequal, and the economic returns to education are lower for immigrants than for non-immigrants. While previous studies have shown that among immigrants education is related to health in the form of a “flat gradient”, interpretations of this evidence have been mixed (Acevedo-Garcia et al., 2007; Balistreri and Van Hook, 2009; Riosmena and Dennis, 2012). Several potential mechanisms that may explain the flat social gradient in health among immigrants have been proposed: factors related to conditions in the country of origin, or "gradient importation"; factors related to emigration and return migration, or "SES-graded health selection"; and acculturation and protection mechanisms in the receiving countries, or "SES-graded acculturation" (Riosmena and Dennis, 2012, p. 97). However, existing evidence has primarily focused on the flat relationship between education and health without fully addressing how education impacts the dynamics of immigrant health over the life-course. We argue that a longitudinal approach will ensure a deeper understanding of the relationship between education and immigrant health and reveal whether health trajectories vary significantly based on educational attainment. We believe this adds valuable evidence to the literature, going beyond a simple association between education and health.

Given the evidence of a “flat-gradient” of education on immigrants’ health found in previous literature, it is important to consider alternative measures of socio-economic status. Income is a strong predictor of health, as it has a direct impact on individuals’ access to the material resources they need for their biological survival and influences their social involvement and environment, which, in turn, shape the conditions that support good health (Marmot, 2002). Studying the role of income and education on the differential health trajectories of immigrants and non-immigrants can offer insights on the mechanisms behind the flat gradient of education on immigrants’ health.

Research on health and mortality by marital status consistently shows that unmarried individuals typically experience poorer health and higher mortality risk compared to their married counterparts, with men being particularly vulnerable (Robards et al., 2012). As marital status patterns are different between immigrants and non-immigrants, and can vary within the immigrant population (Andersson et al., 2015), it is crucial to consider the role that marital status can play in shaping health differentials between immigrants and non-immigrants over the life-course.

This paper advances our understanding of the unhealthy ageing of immigrants, taking Germany as an important case study, given its role in the current migration history of the European context. Germany's immigration history is marked by important waves of migration, which have shaped its current cultural and social landscape. Modern immigration, with the first substantial influxes, took place after World War II including returning Germans prisoners of war, refugees of German descent and from the former German Democratic Republic (GDR) (Bosswick and Borkert, 2011). In the second half of the 50s Germany started the so-called "Gastarbeiter" (guest workers) program. Large recruitments of immigrant workers from Turkey, Italy, Spain, Greece, and other countries to support its booming economy were put in place (Bosswick and Borkert, 2011). More recently, the fall of the Berlin Wall in 1989 led to increased immigration from Eastern Europe, while the 2015 refugee crisis brought a surge of asylum seekers, particularly from Syria (Ayoub, 2023).

Germany has a healthcare system characterized by universal coverage, with a mix of public and private providers. Funded primarily through compulsory health insurance, the system ensures that residents, including immigrants, have access to complete medical care (Razum et al., 2016). Immigrants in Germany, whether long-term residents or asylum seekers, are generally entitled to healthcare services, although access may vary based on their legal status. Asylum seekers receive basic health coverage, including for acute conditions and during pregnancy and childbirth, while those with permanent residency enjoy full benefits (Razum et al., 2016). A recent systematic review found that, compared to non-immigrants, immigrants have lower utilization rates for specialist care, medication, therapist consultations, counselling, and disease prevention services, such as early cancer screenings, childhood prevention programs, and oral health check-ups. However, there are inconsistent trends observed in the case of visits to general practitioners or paediatricians, inpatient care, and vaccinations (Klein and von dem Knesebeck, 2018).

## Aims and research questions

The first aim of this paper is to describe how the healthy ageing trajectories of immigrants and non-immigrants differ. Our first research question is as follows: (*RQ 1*) Compared to non-immigrants, are immigrants ageing in poorer self-perceived health, and with a higher probability of having a physical limitation?

The second aim is to test whether education plays a protecting role in the development of the health gap between immigrants and non-immigrants with age. We ask the following questions: (*RQ 2*) Does higher education play a role in mitigating the differential health trajectories of immigrants and non-immigrants as they age? Is higher education associated with a narrowing of the health gap between immigrants and non-immigrants?

The third aim is to consider two additional socio-economic layers: income and marital status. We include income in our analysis in order to test whether the potential immigrant health disadvantage by education persists or is reduced when immigrants and non-immigrants have equal income levels. Testing this association also sheds light on the question of whether the unclear association between health and education observed among immigrants (Acevedo-Garcia et al., 2007; Balistreri and Van Hook, 2009) can be explained by immigrants having lower economic returns to education. We include marital status, as marriage is a strong protective factor for health (Dupre et al., 2009; Rendall et al., 2011). However, the marriage patterns of immigrants are very different from those of non-immigrants (Andersson et al., 2015). We therefore ask the following question: (*RQ 3*) Does being married and having a high socio-economic status protect immigrants from experiencing a more rapid health decline?

The fourth aim is to examine whether there are sex differences in the abovementioned mechanisms. Thus, we ask the following question: (*RQ 4*) Is the hypothesised differential health decline between immigrants and non-immigrants especially pronounced among women?

Finally, as a sensitivity check, we test whether the relationship between age and health depends not only on immigration background, but also on the specific country of birth (results available in the online supplementary material).

## Data and methods

### Data

We use data from the German Socio-Economic Panel (G-SOEP), a representative longitudinal study of private households including German nationals, foreign citizens, and immigrants to Germany. Topics covered by the survey include household composition, occupational biographies, employment, earnings, health and satisfaction indicators. We use waves 1994–2019 to study self-rated health and waves 2002–2019 to study disability, as the latter is not available in waves before 2002. We focus on the population aged 30–80 years. We do not study ages above 80 because of limited sample size and in order to limit the effect of the so-called “salmon bias”. The salmon bias hypothesis (Pablos-Mendez, 1994) suggests that foreign-born individuals tend to return to their countries of origin in significant numbers when their health deteriorates, which is more likely to occur with ageing. This selective emigration leads to lower reported rates of poor health and mortality for the immigrant population than would be seen if the health and mortality of these return migrants were included in the statistics (Turra and Elo, 2008).

We exclude from our sample individuals with the following characteristics: individuals who were under age 18 at immigration, or second-generation immigrants, in order to avoid heterogeneity in health selectivity by generation that could bias our results (Loi et al., 2021); individuals who migrated to Germany less than 10 years prior to entering the study in order to avoid health selectivity of recently arrived immigrants due to the healthy immigrant effect (Loi and Hale, 2019); individuals with missing information on the two outcomes, self-rated health and disability, and on the covariates of interest, education, marital status, and income. The final sample used to study self-rated health consisted of 57,401 individuals: 6259 immigrants (3026 men and 3233 women) and 51,142 non-immigrants (24,739 men and 26,403 women). The sample used for studying disability consisted of 41,934 individuals: 4477 immigrants (2079 men and 2398 women) and 37,466 non-immigrants (17,809 men and 19,657 women) (Table 1). The distribution of missing values in the final sample is qualitatively similar between immigrants and non-immigrants (Table A5 in the online supplementary material).

**Table 1:** Descriptive statistics of the samples. Individuals aged 30–80, Germany, SOEP waves 1994–2019 for self-rated health, and waves 2002-2019 for disability.

	Outcome: self-rated health				Outcome: disability			
	Non-immigrants		Immigrants		Non-immigrants		Immigrants	
	**Men, N = 24,739**	**Women, N = 26,403**	**Men, N = 3026**	**Women, N = 3233**	**Men, N = 17,809**	**Women, N = 19,657**	**Men, N = 2079**	**Women, N = 2398**
**Mean age**	47.4 (13.7)	46.7 (13.9)	48.5 (10.6)	46.4 (10.6)	49.4 (13.6)	48.7 (13.7)	50.7 (11.5)	48.3 (11.2)
**Age group**								
30–40	9354 (37.8%)	10,786 (40.9%)	808 (26.7%)	1130 (35.0%)	5637 (31.7%)	6590 (33.5%)	476 (22.9%)	699 (29.1%)
40–50	5665 (22.9%)	6050 (22.9%)	1012 (33.4%)	1091 (33.7%)	4354 (24.4%)	5045 (25.7%)	614 (29.5%)	773 (32.2%)
50–60	4661 (18.8%)	4363 (16.5%)	754 (24.9%)	631 (19.5%)	3456 (19.4%)	3508 (17.8%)	526 (25.3%)	527 (22.0%)
60+	5059 (20.4%)	5204 (19.7%)	452 (14.9%)	381 (11.8%)	4362 (24.5%)	4514 (23.0%)	463 (22.3%)	399 (16.6%)
**Education**								
Primary	1421 (5.7%)	3530 (13.4%)	1117 (36.9%)	1333 (41.2%)	995 (5.6%)	2365 (12.0%)	701 (33.7%)	911 (38.0%)
Secondary	15,447 (62.4%)	16,617 (62.9%)	1309 (43.3%)	1131 (35.0%)	11,072 (62.2%)	12,508 (63.6%)	929 (44.7%)	872 (36.4%)
Tertiary	7871 (31.8%)	6256 (23.7%)	600 (19.8%)	769 (23.8%)	5742 (32.2%)	4784 (24.3%)	449 (21.6%)	615 (25.6%)
**Income**								
Low	6676 (27.0%)	8899 (33.7%)	1081 (35.7%)	1178 (36.4%)	5172 (29.0%)	6744 (34.3%)	799 (38.4%)	938 (39.1%)
Medium	8168 (33.0%)	8406 (31.8%)	1167 (38.6%)	1198 (37.1%)	6162 (34.6%)	6542 (33.3%)	854 (41.1%)	901 (37.6%)
High	9895 (40.0%)	9098 (34.5%)	778 (25.7%)	857 (26.5%)	6475 (36.4%)	6371 (32.4%)	426 (20.5%)	559 (23.3%)
**Marital status**								
Unmarried	7744 (31.3%)	9670 (36.6%)	418 (13.8%)	715 (22.1%)	5552 (31.2%)	7188 (36.6%)	314 (15.1%)	579 (24.1%)
Married	16,995 (68.7%)	16,733 (63.4%)	2608 (86.2%)	2518 (77.9%)	12,257 (68.8%)	12,469 (63.4%)	1765 (84.9%)	1819 (75.9%)
**Mean self-rated health**	3.6 (0.9)	3.5 (1.0)	3.4 (1.1)	3.3 (1.0)	–	–	–	–
**Disability**								
Yes	–	–	–	–	6221 (34.9%)	8041 (40.9%)	842 (40.5%)	1111 (46.3%)
**Disability change**								
No disability in all waves	–	–	–	–	8073 (45.3%)	7682 (39.1%)	905 (43.5%)	903 (37.7%)
Change from no to yes	–	–	–	–	5333 (29.9%)	6287 (32.0%)	543 (26.1%)	644 (26.9%)
Disability in all waves	–	–	–	–	4403 (24.7%)	5688 (28.9%)	631 (30.4%)	851 (35.5%)
**Countries of birth**								
Germany	24,739 (100.0%)	26,403 (100.0%)	–	–	17,809 (100.0%)	19,657 (100.0%)	–	–
Italy	–	–	208 (6.9%)	146 (4.5%)	–	–	136 (6.5%)	95 (4.0%)
Poland	–	–	260 (8.6%)	388 (12.0%)	–	–	205 (9.9%)	307 (12.8%)
Turkey	–	–	428 (14.1%)	354 (10.9%)	–	–	261 (12.6%)	248 (10.3%)
Others	–	–	2130 (70.4%)	2345 (72.5%)	–	–	1477 (71.0%)	1748 (72.9%)

### Outcome measures

To ensure comparability when studying groups with different cultures and disease patterns, it is essential to choose appropriate health indicators. Additionally, reflecting on the appropriateness of the comparison is critical (Braveman et al., 2010; Burgard and Chen, 2014). To ensure comparability we include two indicators of morbidity that define different dimensions of health: self-rated health and disability. Self-rated health is measured on a scale from 0 to 4 (very good, good, fair, poor, very poor), and we recode it such that the highest value indicates worse health, to ensure comparability with disability. The link between the biological aging process and the deterioration of self-rated health has been widely observed, and reflects the connection between an individual's perception of their health and the actual physiological changes in their body. Although self-rated health is a subjective measure, it is accurate in predicting biological health outcomes, including morbidity and mortality (Cislaghi and Cislaghi, 2019). Disability is a more objective indicator, and it is less likely than self-rated health to be subjected to cultural bias. Disability is calculated using the following items of the Activities of Daily Living (ADL) scale: having trouble getting out of bed, having trouble shopping, having trouble doing housework (all collected every year since 1985), having trouble dressing (collected every year since 1991), and having trouble climbing stairs (collected every two years since 2002). The outcome variable takes value 1 if the individual has one or more limitations in the abovementioned dimensions, and takes value 0 if the individual has no limitations. A more refined categorization of disability was not possible due to the limited sample size. For both outcomes, we interpret the results in the same direction: i.e., the higher the value, the worse the health outcome. The comparison of the results using the two indicators provides a test of the robustness of the results between subjective and objective measures of morbidity.

### Main exposures and covariates

Our main exposure of interest is the interaction between age and immigration background. Immigration background is a binary variable that takes value 0 for non-immigrants and value 1 for immigrants. Non-immigrants are individuals born in Germany while immigrants are individuals born outside of Germany, who crossed an international border for any reason, and who are regular residents in Germany. In order to consider the heterogeneity in the immigrant population, we include supplementary analyses that consider the main countries of birth of the immigrant population in Germany. The age of the individuals included in our analysis ranges from 30 to 80. Age is included in the model as a continuous variable, and modelled by using a spline function with three components, which relies less on pre-imposed functional specifications, and allows for more flexibility (de Boor, 1980). Education is defined using three categories: less than high school, high school, and higher than high school. As we do not have information about the country where the degree was obtained (Germany vs. other countries), we use this broad definition of education in order to limit issues of comparability across different educational systems. Still, it is important to point out that levels of education might have slightly different qualitative meanings across different societies, and that our strategy does not fully prevent a certain amount of bias due to these differences (we discuss this issue further in the limitations section). As a further step, we include marital status and income as control variables. These two controls are measured at the same time as the health outcomes. We acknowledge that this approach limits our ability to draw causal inference, which is nevertheless beyond the scope of this study.

## Method

We describe and explain how the health trajectories of immigrants and non-immigrants differ with age applying random-effects regression models. We treat self-rated health as a continuous variable, ranging from 0 to 4, and disability as a dichotomous variable with values 0 = without disability, and 1 = with disability. In order to account for the bias due to loss to follow-up, and the potential salmon bias, we apply inverse probability weighting (IPW) techniques. Individuals are weighted by the inverse of their probability of participating in the study. This probability is estimated using each individual's characteristics, including age, education, marital status, and income. The use of IPW implies that an individual with a high probability of response is given a lower weight in the analysis (Metten et al., 2022).

First, we test whether the relationship between health and age is different between immigrants and non-immigrants, including an interaction term between age and immigration background. This is modelled with the equation

(1) $$\begin{array}{c} Y_{\mathrm{it}}=\alpha_{i}+\sum_{j=1}^{3}\beta_{1j}\mathrm{bs}\left(\mathrm{age},\;j\right)+\beta_{2}\;I\left(\mathrm{mig}\right)+\sum_{j=1}^{3}\beta_{3j}\mathrm{bs}\left(\mathrm{age},\;j\right)\times I\left(\mathrm{mig}\right)+\varepsilon \;\; \end{array}$$

where $Y_{it}$ is the health outcome of individual i at wave t, $\alpha_{i}$ is individual random effect, $\sum_{j=1}^{3}\beta_{1i}bs\left(age,\;j\right)$ is the sum of the three age components modelled with a spline function, $\beta_{2\;}I\left(mig\right)$ is immigration background, and ε is the error term. We stratify model 1 (Eq. (1)) by sex in order to explore sex differences in the health gap between immigrants and non-immigrants with age.

Second, we stratify model 1 (Eq. (1)) by educational level and sex in order to test whether the differential weathering process is buffered by education. We test the hypothesis that at higher levels of education, differences in the health trajectories of immigrants and non-immigrants are attenuated.

Third, we include marital status $\beta_{4\;}I\left(marstat\right)$ and income $\beta_{5\;}I\left(income\right)$ and an interaction term between marital status and immigration background, and between income and immigration background (Eq. (2)), to account for the differential relationship between these two characteristics and health, depending on whether the individual is an immigrant or a non-immigrant.

(2) $$Y_{it}=\alpha_{i}+\sum_{j=1}^{3}\beta_{1j}bs\left(age,\;j\right)+\beta_{2\;}I\left(mig\right)+\sum_{j=1}^{3}\beta_{3i}bs\left(age,\;j\right)\;\times I\left(mig\right)+\beta_{4\;}I\left(marstat\right)+\beta_{5\;}I\left(income\right)+\beta_{4\;}I\left(marstat\right)\times \beta_{5\;}I\left(income\right)+\;\varepsilon \;\;$$

### Sensitivity checks

First, we run the models using pooled Ordinary Least Squares regression (OLS); second, we use pooled generalized linear models for self-rated health and disability respectively; third, we run the models using individual fixed-effects models which estimate changes within individuals, addressing any potential unobserved confounding factors, assuming these factors remain constant over time (Allison, 2009). In the fixed-effects models, the immigration background variable, which is time constant, was included via an interaction between age (time-varying) and migration background (time constant) (see Allison 2009 for the inclusion of time constant variables in fixed-effects models). Fourth, we include country of birth as a stratification factor to test whether the relationship between age and health differs depending on the specific country of birth. Thus, we test model 1 (Eq. (1)) stratified by the three largest immigrant groups: born in Turkey, Italy, Poland, or other countries. Fifth, in the online Appendix, we provide results on the unweighted models to show the extent of the bias correction (Figs. A7 and A8). For self-rated health, the weighted models adjust for health selection at younger ages, resulting in a smaller gap between immigrants and non-immigrants at older ages. Regarding disability, the weighted models also account for health selection at young ages, showing narrower health gaps between immigrants and non-immigrants among men, but wider gaps among women.

Additionally, self-rated health was modelled as a continuous variable, ranging from 0 to 4, despite being ordinal. This decision was taken in order to estimate health trajectories over age, which requires calculating average health values, something that cannot be achieved when treating self-rated health as an ordinal variable. We recognize that this approach assumes the five categories of self-rated health are equidistant, which may not be accurate. To address this, we conduct further sensitivity checks by estimating the same models using logit and probit models and comparing the results, which are highly consistent with the estimates presented in this manuscript (results provided in Fig. A9 in the online appendix).

## Results

We estimate the same set of models for the two outcomes, self-rated health and disability. Table 1 describes the two samples. Immigrants have a younger age structure than non-immigrants and are more likely to be married, which are protective factors for health. However, immigrants of both sexes are overrepresented in the lowest educated and lowest income groups and are underrepresented in the highest education and income groups, which are strong risk factors for poor health. Immigrant women with tertiary education represent an exception, as their share in the high educated group is more similar to that of non-immigrant women, compared with the differential by migration background among men. The average values of self-rated health are similar for immigrants and non-immigrants and for both men and women, while the proportion of individuals with disability is higher among immigrants than among non-immigrants of both sexes.

### Weathering

**Fig. 1:**
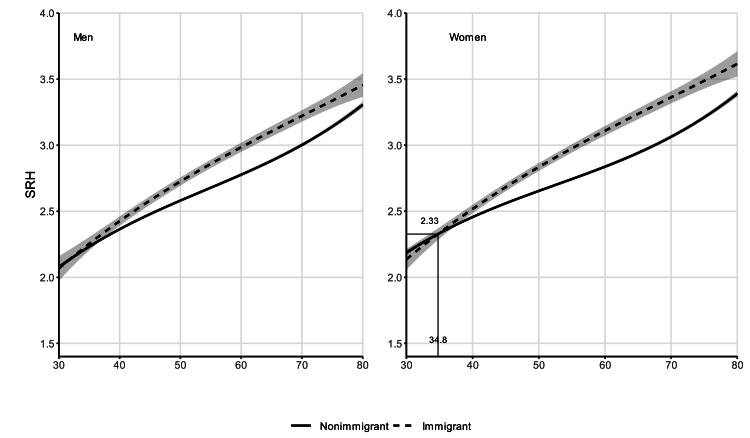
Self-rated health trajectories by age and immigration background, stratified by sex, from random-effects models. Individuals aged 30–80, Germany, SOEP waves 1994–2019 (full estimates in Tables A1–A4). Models weighted with inverse probability weighting.

**Fig. 2:**
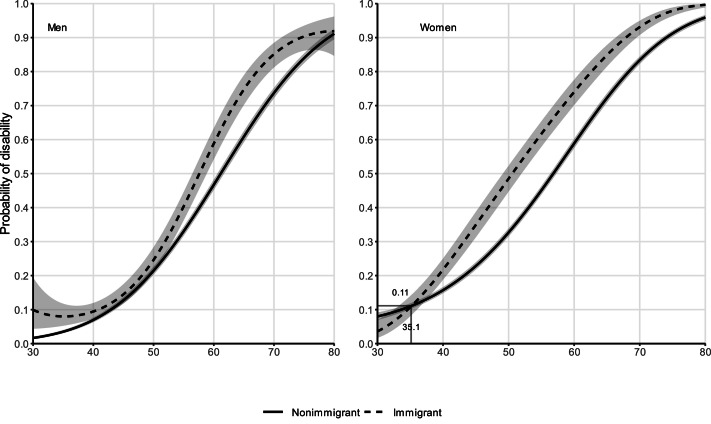
Disability trajectories by age and immigration background, stratified by sex, from random-effects models. Individuals aged 30–80, Germany, SOEP waves 2002-2019 (full estimates in Tables A1–A4). Models weighted with inverse probability weighting.

**Fig. 3:**
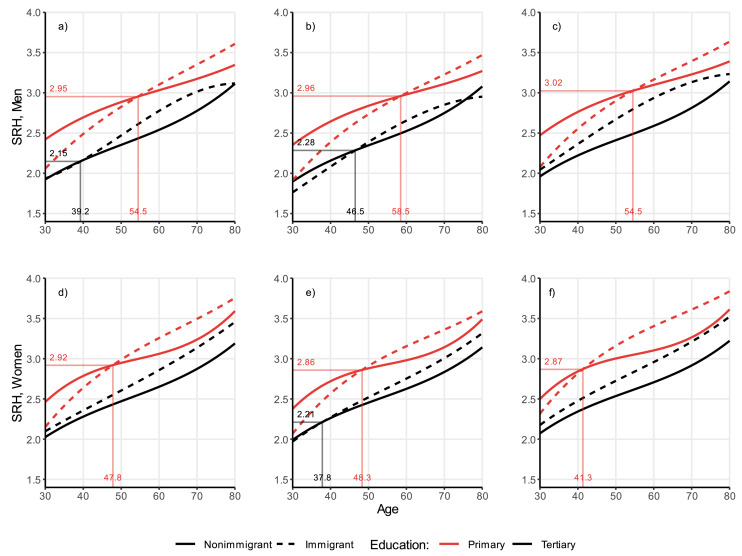
Self-rated health trajectories by age and immigration background, stratified by sex and education, from random-effects models. Individuals aged 30–80, Germany, SOEP waves 1994–2019 (full estimates in Tables A1–A4). Models weighted with inverse probability weighting.

Notes: Panel A: men, total; panel B: married men with high income; panel C: unmarried men with low income; panel D: women, total; panel E: married women with high income; panel F: unmarried women with low income.

**Fig. 4:**
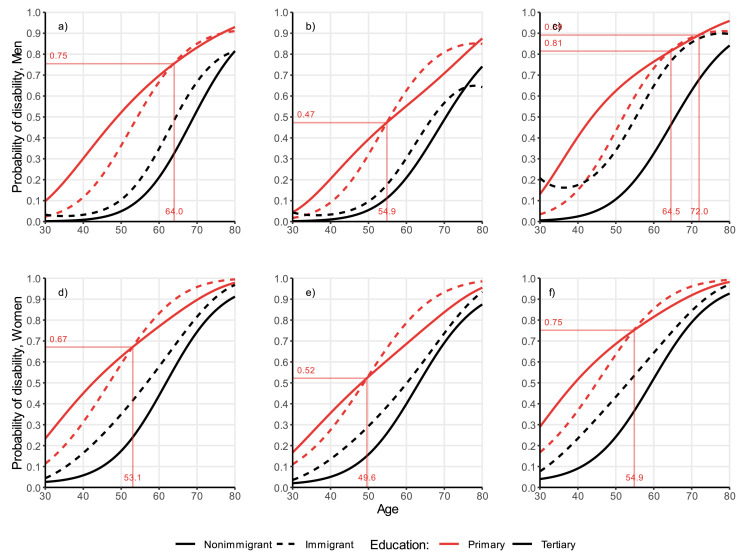
Disability trajectories by age and immigration background, stratified by sex and education, from random-effects models. Individuals aged 30–80, Germany, SOEP waves 2002-2019 (full estimates in Tables A1–A4). Models weighted with inverse probability weighting.

Notes: Panel A: men, total; panel B: married men with high income; panel C: unmarried men with low income; panel D: women, total; panel E: married women with high income; panel F: unmarried women with low income.

We present and discuss the results from the models graphically (Fig. 1, Fig. 2, Fig. 3, Fig. 4), and we include the full estimates of each model in the online supplementary material Tables A1–A4. All models presented here are weighted using inverse probability weighting in order to account for the loss to follow-up, and the so-called “salmon bias” (Abraído-Lanza et al., 1999). In Fig. 1, we show the self-rated health trajectory by age, immigration background, and sex; and in Fig. 2, we show the same trajectory for disability. Solid lines indicate non-immigrants, and dotted lines indicate immigrants. For both outcomes, we observe similar health levels among immigrants and non-immigrants at age 30, and a crossover occurring at ages below 40 among women (self-rated health: 34.8; disability: 35.1). We do not observe a clear crossover age among men: i.e., compared to non-immigrants, immigrant men do not have a health advantage at younger ages (30 to 45). As age increases, the health of immigrants declines faster than that of non-immigrants of both sexes and for both health outcomes. In the case of disability, among men we observe a trend towards convergence between immigrants and non-immigrants at the oldest ages. We still observe significant differences in the probability of having a disability between female immigrants and female non-immigrants at age 80.

### The role of education

In Fig. 3, we show: 1) the differential self-rated health decline by education (panels A for men and D for women); 2) the same patterns for individuals who are the most advantaged: i.e., who are married and have a high income (panels B for men and E for women); and for individuals who are the most disadvantaged: i.e., who are unmarried and have a low income (panels C for men and F for women). Education is shown by different line colours: red for primary education and black for tertiary education (for ease of comparison, we do not plot the results for secondary education, although its effect is estimated in the models, and the results are included in Appendix Tables A1–A4). Although the results come from stratified models, we plot them in the same figure to facilitate the comparison. In Fig. 4, we show the same figures for the outcome disability.

As expected, when stratifying for education (Fig. 3, panels A and D), we observe differences in the overall levels of the self-rated health trajectories: i.e., we find that across the whole age span, self-rated health is poorer among the lowest educated and is better among the highest educated. Most importantly, we find that the health gaps between immigrants and non-immigrants differ between the two education groups. Among the lowest educated (red lines), we observe wide health gaps at younger ages with an immigrant advantage, and wide health gaps at older ages, with an immigrant disadvantage. Among the highest educated individuals (black lines), immigrant men (panel A) have similar health levels compared to non-immigrant men until age 39.2, when they start a steeper health decline, and a convergence at older ages. Immigrant women (panel D), at higher levels of education (black lines) display consistent health disadvantage throughout the life-course.

For disability (Fig. 4, panels A and D), we observe that, in line with the patterns found for self-rated health, the lowest educated have a higher probability of having a disability across the whole age span, compared to the highest educated, irrespective of the migration background. Focusing on the disability gap between immigrants and non-immigrants, among the highest educated, immigrants are consistently disadvantaged, while among the lowest educated immigrants have a health advantage at younger ages, which converges to non-immigrants' levels at older ages.

### The role of income and marital status

When estimating the associations in specific marital status/income groups, we are interested in comparing the differences between immigrants and non-immigrants in the self-rated health (Fig. 3) and disability trajectories (Fig. 4) of the most advantaged socio-economic group (married individuals with high income, panels B for men and panels E for women in both figures) and the most disadvantaged group (unmarried individuals with low income, panels C for men and panels F for women in both figures). Across the whole age span, we observe lower overall estimates of self-rated health (and thus better health) among the most advantaged individuals (Fig. 3 panels B and E) than among the most disadvantaged groups (C and F). However, the differentials between immigrants and non-immigrants by education are very consistent with the general pattern described in panel A for men, and B for women, indicating that income and marital status appear to be secondary to other unobserved determinants.

### Ages at crossover

For self-rated health, among men with lower education, the age at crossover is 54.5 (panel A), 58.5 for the most socio-economically advantaged group (married with high income), and 54.5 for the most disadvantaged group (unmarried with low income); among men with higher education, the age at crossover is 39.2 (panel A), 46.5 for the most socio-economically advantaged group, while no age at crossover is observed for the most socio-economically disadvantaged group, due to a consistent health disadvantage of immigrants. The younger age at crossover for the most disadvantaged groups is an indication that immigrants’ accelerated ageing is further exacerbated by a higher socio-economic vulnerability.

For disability, when looking at the ages at crossover, we observe some notable differences with self-rated health. Since among the highest educated population immigrants are at a consistent health disadvantage through the life-course, no age at crossover is estimated; while for the lowest educated, the age at crossover is higher for the most socio-economically disadvantaged group (64.5, unmarried, with low income) compared to the most advantaged group (54.9). However, in this case the age at crossover differences are primarily due to shifts in the age patterns among non-immigrants. Importantly, the age pattern among immigrants remains relatively consistent across the most and least socio-economically disadvantaged groups, suggesting a weaker role in the protective effects of income and marital status in shaping disability trajectories among immigrants.

### Sensitivity checks

Results of the sensitivity checks are available in the online supplementary material. First, we run the same models using pooled OLS regressions (Figs. A1 and A2). Second, we test the same models using individual-fixed effects models (Figs. A3 and A4). Fixed-effects models, estimating changes within individuals, inherently address any potential unobserved confounding factors, assuming these factors remain consistent over time (Allison, 2009). The overall patterns observed with random-effects models, presented here, are consistent with results from both pooled OLS regressions and fixed-effects models, presented in the supplementary material. Third, we stratify the models by country of birth (Figs. A5 and A6), focusing on the largest immigrant groups: Turkey, Italy, Poland, or other countries. Due to sample size problems, we can only show the overall health trajectories by age, but we include education as a control. The overall pattern of a more rapid health deterioration by age is observed for all subgroups, with some differences by countries in the size of the gap compared to non-immigrants. For both outcomes, the fastest health declines and the largest differences compared to non-immigrants are observed for immigrants born in Turkey across all ages. A very rapid increase in the probability of having a disability is observed for immigrant women born in Italy. Men who immigrated from Italy show a pattern of self-rated health selection at exit that results in a slower health decline at older ages, particularly starting from around age 65 (around pension age). This finding is most likely attributable to a high proportion of Italian-origin men migrating back to their origin country upon retirement. Immigrants from Poland, display a pattern that is very similar to the average trend among immigrants, with a steeper health decline being observed at older ages (70+), except in the case of disability among men, for whom a slower health decline at older ages (70+) is found.

## Discussion

In this paper, we study the joint role of age and sex on the weathering of immigrants and non-immigrants, and investigate the protective role of education and socio-economic conditions. Weathering is the process through which cumulative and stress-mediated wear and tear on cellular integrity leads to accelerated ageing, the early onset of chronic diseases, and excess mortality among marginalised groups, including immigrants (Geronimus, 1992, 1996; Jones et al., 2019). This process can be interpreted as a physical consequence of the social inequality experienced by immigrants in the receiving context. Since age has been shown to be not only an indicator of biological development, but also a reflection of the ways in which social inequality, discrimination, or bias in exposures to psychosocial or environmental hazards may differentially affect the health of certain groups (Wildsmith, 2002), we argue that this paper shows how the physical consequences of social inequalities in the form of health outcomes differ between non-immigrants and immigrants.

First, we observe that immigrants are ageing in poorer self-perceived health, and with a higher probability of having a physical limitation. We show that at younger ages, immigrants have either the same level or better health than non-immigrants, irrespective of their sex and education. However, the health of immigrants deteriorates at a faster pace over the life-course, which results in a clear and strong immigrant health disadvantage at older ages, with widening disparities as age increases. Second, we find evidence that low education is linked to higher selection into better health at younger ages among immigrants compared to non-immigrants, but this selection vanishes rapidly over the life-course resulting in widening gaps at older ages. These gaps at older ages are relatively similar irrespective of education, indicating that the health decline among the lowest educated immigrants is steeper compared to that of the highest educated immigrants.

We also find that being married and having a high income provides no further protective effect to immigrants, suggesting that other unobserved factors might be at play in determining their faster health deterioration over the life-course. Finally, we find that there are sex differences in the differential weathering process between immigrants and non-immigrants. Among women, we observe wider disparities between immigrants and non-immigrants with age, indicating an exacerbated vulnerability of immigrant women, for both health outcomes. This is reflected in the analysis of the age at crossover which consistently occurs earlier among women than it does among men, for both outcomes, and at different levels of education.

This study is not without limitations. First, as was mentioned earlier, we use broad education categories, as we do not have access to information about the country where the degree was obtained. A given educational level may have a different qualitative meaning across different cultures, and our analytical strategy does not fully control for this bias. However, some considerations reassure us that our results are robust. Our results by education go in the expected direction, and are corroborated by previous literature showing that inequalities are wider at lower levels of education. If our classification approach suffered from misclassification bias, our results should have reflected unexpected patterns. Thus, we are confident that this categorisation, although crude, is efficient in explaining the observed health disparities between immigrants and non-immigrants. Also, the analyses are further adjusted by income, which is a solid socio-economic status indicator and a strong predictor of health.

Second, we cannot fully control for the out-migration bias; i.e., the so–called “salmon bias” (Abraído-Lanza et al., 1999), or healthy remigration (Wallace and Kulu, 2014). In fact, information on the health status of individuals who have left Germany to go back to their origin countries, or who have emigrated to a third country, is not available. There is conflicting evidence regarding how health influences return migration that supports both selection for poor health (Abraído-Lanza et al., 1999; Riosmena et al., 2013) and selection for good health (Sander, 2008). Immigrants may return to their home country when they are gravely ill, when they reach pension age, or alternatively, when they are healthy. However, further data exploration (not shown), reveal that in our data poor health is linked to a higher probability of loss to follow-up. It therefore appears that out-migration produces a bias towards an underestimation of poor health. Therefore, we can safely assume that our results would reflect even stronger inequalities, had we had complete information on the health conditions of individuals who out-migrated.

Third, due to sample size issues, we could not fully explain the observed patterns of weathering by country of birth. In particular, we were unable to run stratified models by education, socio-economic conditions, and by country of birth. Thus, we could not test whether marital status and income contribute to explain the differences in healthy ageing trajectories across specific origins. However, we do show the overall weathering process by country of birth, controlling for education, finding similar patterns across origins, albeit with some differences in the magnitude of the gap by countries, with non-immigrants.

Fourth, specific to the analyses on self-rated health, it is important to note that immigrants may consider their non-migrant counterparts in their country of birth as their health reference group. This reference group may have worse health on average, compared to non-immigrant Germans, who may have better health. However, we found very similar patterns for self-rated health and disability - a more objective indicator - in the observed health gaps, which makes us confident about the robustness of the results.

Fifth, our analyses did not include time or duration of stay, which are closely linked to changes in immigrant composition and key determinants of health profiles, to avoid issues associated with age-period-cohort effects. To address potential biases arising from this limitation we implemented three strategies: we focused only on first-generation immigrants to reduce heterogeneity in health selectivity by generation that could skew our results (Loi et al., 2021); we limited our analysis to individuals who have resided in Germany for over ten years to avoid health selectivity related to the healthy immigrant effect among more recently arrived individuals (Loi and Hale, 2019); we conducted sensitivity analyses based on the most numerous groups of immigrants by countries of origin to account for variations in the immigrant population's composition.

Sixth, the health of ageing immigrants may deteriorate due to other risk factors that were not measured in the data, and that we could not consider in our analyses. A further step towards understanding why immigrants age in poorer health may involve taking discrimination pathways into account, including discrimination in health care access.

Finally, self-rated health was modelled as a continuous variable, ranging from 0 to 4, despite being an ordinal variable. We acknowledge that this approach assumes the five categories are equidistant, which may not be the case. However, following several sensitivity analyses, including the estimation of identical models using ordinal logistic regression, which generated results nearly identical to our own, we chose to model self-rated health as a continuous variable. This method is more appropriate for estimating health profiles over ages. A comparison of the estimated age profiles using the OLS and ordinal logistic regression approaches is provided in Fig. A9 (online appendix). As shown, the estimated values from both methods are closely aligned. Furthermore, when comparing the self-rated health levels predicted by a linear model with those predicted by an ordinal logistic regression (results not shown), the correlation coefficient was found to be 0.99.

Despite these limitations, this paper, taking a longitudinal life-course approach, provides a novel contribution to the literature on how structural factors, both separately and combined, can produce and exacerbate health inequalities between immigrants and non-immigrants with age. Our results offer important novel scientific knowledge, that has several significant policy implications. We emphasize the importance to address the health needs of immigrants as a marginalized group. In fact, that disparities persist after controlling for several measures of socio-economic conditions, suggests that these disparities are exacerbated by systemic inequities. That is reflected in the evidence that immigrant women have heightened vulnerability to unhealthy ageing. Policies must target the root causes of such systemic inequities, include initiatives to reduce poverty, improve access to quality education, promote equitable housing and employment opportunities for immigrants that match their educational levels. By addressing these fundamental issues, the overall stress burden on marginalized communities and its consequences on health can be alleviated. Moreover, even in contexts with universal health coverage, like European countries, disparities in healthcare access and usage persist among marginalized populations, including immigrants. That calls for the need to implement targeted policies to address inequities in the health care access as well.

The immigrant population in the European context is entering ages at which the risk of developing health frailties becomes higher, and the inequalities in health are exacerbated by ageing. Extending our scientific knowledge by addressing inequalities in healthy ageing between marginalized groups and dominant groups with a life-course perspective is essential to formulate effective policies to prevent immigrants’ health excessive deterioration, and to take tangible steps to improve immigrants' social inclusion.

## CRediT authorship contribution statement

**Silvia Loi:** Writing – original draft, Writing – review & editing, Supervision, Methodology, Funding acquisition, Conceptualization. **Peng Li:** Visualization, Methodology, Formal analysis, Data curation, Conceptualization. **Mikko Myrskylä:** Writing – review & editing, Methodology, Funding acquisition, Conceptualization.

## Declaration of competing interest

The authors declare that they have no known competing financial interests or personal relationships that could have appeared to influence the work reported in this paper.

## Appendix Supplementary materials

Image, application 1
