# Supplementary material for: Unequal weathering: How immigrants’ health advantage vanishes over the life-course
Source: J Migr Health. 2025 Jan 16;11:100303. doi: 10.1016/j.jmh.2025.100303 (PMC11795556; doi:10.1016/j.jmh.2025.100303)
Supplement: Supplementary file 1 [file mmc1.docx]

**Online supplementary material**

Table A1. The relationship between the intersection of age, immigration background, and sex and self-rated health. Estimates from random-effects models. Men aged 30-80, SOEP waves 1994-2019, Germany. Models weighted with inverse probability weighting.

|  | Total population | | Primary education | | Secondary education | | Tertiary education | |
| --- | --- | --- | --- | --- | --- | --- | --- | --- |
|  | M1_tot | M2_tot | M1_pr. | M2_pr. | M1_sec. | M2_sec. | M1_ter. | M2_ter. |
| **Intercept** | 2.077 *** | 2.139 *** | 2.421 *** | 2.472 *** | 2.122 *** | 2.172 *** | 1.924 *** | 1.961 *** |
|  | (0.009) | (0.010) | (0.043) | (0.046) | (0.011) | (0.012) | (0.016) | (0.018) |
| **Age components of B-splines** | | | | | | | | |
| BS 1 | 0.566 *** | 0.648 *** | 0.544 *** | 0.588 *** | 0.621 *** | 0.678 *** | 0.473 *** | 0.525 *** |
|  | (0.022) | (0.022) | (0.119) | (0.120) | (0.027) | (0.028) | (0.037) | (0.039) |
| BS 2 | 0.629 *** | 0.628 *** | 0.588 *** | 0.600 *** | 0.703 *** | 0.701 *** | 0.516 *** | 0.512 *** |
|  | (0.018) | (0.018) | (0.099) | (0.099) | (0.022) | (0.022) | (0.029) | (0.029) |
| BS 3 | 1.230 *** | 1.216 *** | 0.926 *** | 0.919 *** | 1.266 *** | 1.250 *** | 1.189 *** | 1.181 *** |
|  | (0.015) | (0.015) | (0.074) | (0.075) | (0.019) | (0.019) | (0.025) | (0.025) |
| **Immigration background (ref. non-immigrant)** | | | | | | | | |
| Immigrant | -0.010 | -0.076 | -0.362 *** | -0.393 *** | 0.008 | -0.082 | 0.012 | 0.083 |
|  | (0.048) | (0.053) | (0.108) | (0.118) | (0.067) | (0.075) | (0.105) | (0.114) |
| **Age components of B-splines x immigration background (ref. BS1, non-immigrant)** | | | | | | | | |
| BS1 x immigrant | 0.091 | 0.174 | 0.270 | 0.338 | 0.083 | 0.153 | -0.187 | 0.004 |
|  | (0.113) | (0.114) | (0.260) | (0.262) | (0.155) | (0.157) | (0.252) | (0.255) |
| BS2 x immigrant | 0.364 *** | 0.368 *** | 0.509 ** | 0.517 ** | 0.121 | 0.131 | 0.646 *** | 0.605 *** |
|  | (0.066) | (0.066) | (0.164) | (0.164) | (0.094) | (0.093) | (0.139) | (0.139) |
| BS 3 x immigrant | 0.158 * | 0.180 * | 0.623 *** | 0.640 *** | 0.093 | 0.116 | -0.009 | 0.008 |
|  | (0.073) | (0.073) | (0.168) | (0.168) | (0.103) | (0.102) | (0.157) | (0.158) |
| **Marital status (ref. unmarried)** | | | | | | | | |
| Married |  | -0.009 |  | -0.056 |  | -0.015 |  | 0.025 * |
|  |  | (0.007) |  | (0.034) |  | (0.008) |  | (0.011) |
| **Income (ref. Low)** |  |  |  |  |  |  |  |  |
| Medium |  | -0.068 *** |  | -0.060 * |  | -0.057 *** |  | -0.044 *** |
|  |  | (0.005) |  | (0.026) |  | (0.006) |  | (0.011) |
| High |  | -0.132 *** |  | -0.063 |  | -0.101 *** |  | -0.089 *** |
|  |  | (0.006) |  | (0.038) |  | (0.008) |  | (0.013) |
| **Immigration background x marital status (ref. non-immigrant, unmarried)** | | | | | | | | |
| Immigrant x married |  | 0.029 |  | 0.020 |  | 0.065 |  | -0.010 |
|  |  | (0.027) |  | (0.065) |  | (0.042) |  | (0.049) |
| **Immigration background x income (ref. non-immigrant, low)** | | | | | | | | |
| Immigrant x medium |  | 0.009 |  | 0.040 |  | 0.021 |  | -0.139 *** |
|  |  | (0.016) |  | (0.039) |  | (0.024) |  | (0.040) |
| Immigrant x high |  | -0.056 ** |  | -0.068 |  | -0.052 |  | -0.207 *** |
|  |  | (0.021) |  | (0.054) |  | (0.031) |  | (0.047) |
| nobs | 196279 | 196279 | 13435 | 13435 | 121059 | 121059 | 61785 | 61785 |
| r.squared | 0.203 | 0.209 | 0.198 | 0.201 | 0.214 | 0.217 | 0.183 | 0.186 |
| adj.r.squared | 0.203 | 0.209 | 0.198 | 0.200 | 0.213 | 0.217 | 0.183 | 0.186 |
| statistic | 10665.793 | 11344.909 | 517.360 | 545.332 | 7042.779 | 7299.572 | 3374.821 | 3507.095 |
| p.value | 0.000 | 0.000 | 0.000 | 0.000 | 0.000 | 0.000 | 0.000 | 0.000 |
| deviance | 81526.928 | 82000.183 | 7385.149 | 7395.534 | 51331.891 | 51474.047 | 22769.966 | 22855.326 |
| df.residual | 196271.000 | 196265.000 | 13427.000 | 13421.000 | 121051.000 | 121045.000 | 61777.000 | 61771.000 |
| nobs.1 | 196279.000 | 196279.000 | 13435.000 | 13435.000 | 121059.000 | 121059.000 | 61785.000 | 61785.000 |
| All continuous predictors are mean-centered and scaled by 1 standard deviation. *** p < 0.001; ** p < 0.01; * p < 0.05. | | | | | | | | |

Table A2. The relationship between the intersection of age, immigration background, and sex and self-rated health. Estimates from random-effects models. Women aged 30-80, SOEP waves 1994-2019, Germany. Models weighted with inverse probability weighting.

|  | Total population | | Primary education | | Secondary education | | Tertiary education | |
| --- | --- | --- | --- | --- | --- | --- | --- | --- |
|  | M1_tot | M2_tot | M1_pr. | M2_pr. | M1_sec. | M2_sec. | M1_ter. | M2_ter. |
| **Intercept** | 2.187 *** | 2.239 *** | 2.462 *** | 2.499 *** | 2.213 *** | 2.262 *** | 2.026 *** | 2.072 *** |
|  | (0.009) | (0.009) | (0.033) | (0.034) | (0.011) | (0.012) | (0.015) | (0.018) |
| **Age components of B-splines** | | | | | | | | |
| BS 1 | 0.540 *** | 0.603 *** | 0.736 *** | 0.791 *** | 0.534 *** | 0.581 *** | 0.505 *** | 0.551 *** |
|  | (0.021) | (0.021) | (0.076) | (0.077) | (0.026) | (0.026) | (0.039) | (0.039) |
| BS 2 | 0.544 *** | 0.530 *** | 0.330 *** | 0.314 *** | 0.549 *** | 0.541 *** | 0.528 *** | 0.527 *** |
|  | (0.017) | (0.017) | (0.051) | (0.051) | (0.022) | (0.022) | (0.035) | (0.035) |
| BS 3 | 1.204 *** | 1.183 *** | 1.129 *** | 1.112 *** | 1.157 *** | 1.139 *** | 1.164 *** | 1.150 *** |
|  | (0.014) | (0.014) | (0.041) | (0.041) | (0.018) | (0.018) | (0.033) | (0.033) |
| **Immigration background (ref. non-immigrant)** | | | | | | | | |
| Immigrant | -0.051 | 0.011 | -0.310 *** | -0.183 * | -0.073 | -0.022 | 0.073 | 0.104 |
|  | (0.041) | (0.045) | (0.075) | (0.082) | (0.066) | (0.073) | (0.081) | (0.088) |
| **Age components of B-splines x immigration background (ref. BS1, non-immigrant)** | | | | | | | | |
| BS1 x immigrant | 0.161 | 0.194 | 0.215 | 0.209 | 0.115 | 0.159 | -0.055 | 0.013 |
|  | (0.100) | (0.101) | (0.178) | (0.180) | (0.165) | (0.167) | (0.207) | (0.208) |
| BS2 x immigrant | 0.502 *** | 0.476 *** | 0.790 *** | 0.776 *** | 0.505 *** | 0.468 *** | 0.251 | 0.234 |
|  | (0.065) | (0.065) | (0.111) | (0.112) | (0.109) | (0.109) | (0.146) | (0.146) |
| BS 3 x immigrant | 0.276 *** | 0.245 *** | 0.475 *** | 0.409 *** | 0.210 | 0.187 | 0.194 | 0.196 |
|  | (0.070) | (0.070) | (0.112) | (0.113) | (0.120) | (0.120) | (0.166) | (0.166) |
| **Marital status (ref. unmarried)** | | | | | | | | |
| Married |  | -0.015 * |  | -0.026 |  | -0.032 *** |  | 0.011 |
|  |  | (0.006) |  | (0.018) |  | (0.008) |  | (0.012) |
| **Income (ref. Low)** | | | | | | | | |
| Medium |  | -0.050 *** |  | -0.040 ** |  | -0.036 *** |  | -0.045 *** |
|  |  | (0.005) |  | (0.015) |  | (0.006) |  | (0.012) |
| High |  | -0.107 *** |  | -0.097 *** |  | -0.080 *** |  | -0.093 *** |
|  |  | (0.006) |  | (0.021) |  | (0.008) |  | (0.013) |
| **Immigration background x marital status (ref. non-immigrant, unmarried)** | | | | | | | | |
| Immigrant x married |  | -0.070 ** |  | -0.138 *** |  | -0.018 |  | -0.047 |
|  |  | (0.024) |  | (0.042) |  | (0.040) |  | (0.045) |
| **Immigration background x income (ref. non-immigrant, low)** | | | | | | | | |
| Immigrant x medium |  | -0.027 |  | -0.010 |  | -0.070 * |  | -0.003 |
|  |  | (0.017) |  | (0.029) |  | (0.027) |  | (0.037) |
| Immigrant x high |  | -0.039 |  | 0.011 |  | -0.077 * |  | -0.077 |
|  |  | (0.021) |  | (0.038) |  | (0.035) |  | (0.045) |
| nobs | 219692 | 219692 | 31025 | 31025 | 133850 | 133850 | 54817 | 54817 |
| r.squared | 0.192 | 0.196 | 0.236 | 0.239 | 0.187 | 0.191 | 0.156 | 0.159 |
| adj.r.squared | 0.192 | 0.196 | 0.236 | 0.238 | 0.187 | 0.190 | 0.156 | 0.159 |
| statistic | 10829.334 | 11433.881 | 1499.986 | 1566.420 | 5944.383 | 6180.682 | 2329.195 | 2439.711 |
| p.value | 0.000 | 0.000 | 0.000 | 0.000 | 0.000 | 0.000 | 0.000 | 0.000 |
| deviance | 97058.717 | 97544.750 | 15254.671 | 15279.533 | 59055.214 | 59229.659 | 22721.750 | 22842.371 |
| df.residual | 219684.000 | 219678.000 | 31017.000 | 31011.000 | 133842.000 | 133836.000 | 54809.000 | 54803.000 |
| nobs.1 | 219692.000 | 219692.000 | 31025.000 | 31025.000 | 133850.000 | 133850.000 | 54817.000 | 54817.000 |
| All continuous predictors are mean-centered and scaled by 1 standard deviation. *** p < 0.001; ** p < 0.01; * p < 0.05. | | | | | | | | |

Table A3. The relationship between the intersection of age, immigration background, and sex and disability. Estimates from random-effects models. Men aged 30-80, SOEP waves 2002-2019, Germany. Models weighted with inverse probability weighting.

|  | Total population | | Primary education | | Secondary education | | Tertiary education | |
| --- | --- | --- | --- | --- | --- | --- | --- | --- |
|  | M1_tot | M2_tot | M1_pr. | M2_pr. | M1_sec. | M2_sec. | M1_ter. | M2_ter. |
| **Intercept** | -2.134 (0.048)*** | -1.897 (0.049)*** | -1.293 (0.148)*** | -1.120 (0.152)*** | -1.935 (0.058)*** | -1.828 (0.060)*** | -2.930 (0.0001)*** | -2.541 (0.099)*** |
| **Age components of B-splines** | | | | | | | | |
| BS 1 | 1.055 (0.110)*** | 1.347 (0.111)*** | 1.506 (0.432)*** | 1.831 (0.436)*** | 1.327 (0.137)*** | 1.436 (0.138)*** | 0.692 (0.0001)*** | 0.782 (0.213)*** |
| BS 2 | 2.304 (0.079)*** | 2.264 (0.079)*** | 1.806 (0.345)*** | 1.608 (0.346)*** | 2.248 (0.097)*** | 2.325 (0.098)*** | 2.554 (0.0001)*** | 2.538 (0.143)*** |
| BS 3 | 3.481 (0.075)*** | 3.399 (0.074)*** | 2.767 (0.278)*** | 2.859 (0.280)*** | 3.474 (0.094)*** | 3.403 (0.094)*** | 3.827 (0.0001)*** | 3.542 (0.132)*** |
| **Immigration background (ref. Non-immigrant)** | | | | | | | | |
| Immigrant | 0.847 (0.220)*** | -0.451 (0.233)+ | -0.684 (0.380)+ | -0.696 (0.403)+ | -0.261 (0.304) | -0.290 (0.336) | 1.091 (0.0001)*** | 1.725 (0.562)** |
| **Age components of B-splines x immigration background (ref. BS1, non-immigrant)** | | | | | | | | |
| BS1 x immigrant | -1.854 (0.526)*** | 0.446 (0.519) | -0.315 (0.959) | -0.685 (0.963) | -0.701 (0.731) | -0.451 (0.738) | -1.382 (0.0001)*** | -1.729 (1.210) |
| BS2 x immigrant | 0.381 (0.279) | 1.245 (0.276)*** | 1.321 (0.566)* | 1.695 (0.568)** | 1.704 (0.396)*** | 1.070 (0.395)** | -0.101 (0.0001)*** | -0.175 (0.599) |
| BS3 x immigrant | -0.798 (0.328)* | 0.496 (0.322) | 0.555 (0.605) | 0.291 (0.602) | 0.348 (0.460) | 0.523 (0.462) | -1.097 (0.0001)*** | -1.467 (0.722)* |
| **Marital status (ref. unmarried)** | | | | | | | | |
| Married |  | -0.024 (0.027) |  | -0.233 (0.105)* |  | -0.009 (0.033) |  | 0.028 (0.053) |
| **Income (ref. Low)** | | | | | | | | |
| Medium |  | -0.191 (0.024)*** |  | -0.140 (0.097) |  | -0.134 (0.028)*** |  | -0.170 (0.056)** |
| High |  | -0.484 (0.028)*** |  | -0.355 (0.137)** |  | -0.285 (0.034)*** |  | -0.382 (0.060)*** |
| **Immigration background x marital status (ref. non-immigrant, unmarried)** | | | | | | | | |
| Immigrant x married |  | 0.136 (0.107) |  | 0.123 (0.206) |  | 0.262 (0.160) |  | -0.164 (0.216) |
| **Immigration background x income (ref. non-immigrant, low)** | | | | | | | | |
| Immigrant x medium |  | -0.152 (0.075)* |  | 0.038 (0.151) |  | -0.182 (0.106)+ |  | -0.529 (0.189)** |
| Immigrant x high |  | -0.074 (0.095) |  | 0.165 (0.208) |  | -0.276 (0.142)+ |  | -0.376 (0.215)+ |
| SD (Intercept pid) | 1.434 | 1.394 | 1.225 | 1.212 | 1.370 | 1.357 | 1.660 | 1.538 |
| Num.Obs. | 71746 | 71746 | 4524 | 4524 | 43564 | 43564 | 23658 | 23658 |
| R2 Marg. | 0.223 | 0.237 | 0.216 | 0.222 | 0.228 | 0.236 | 0.228 | 0.235 |
| R2 Cond. | 0.746 | 0.741 | 0.687 | 0.685 | 0.732 | 0.731 | 0.794 | 0.773 |
| AIC | 78449.3 | 78091.7 | 5612.4 | 5605.9 | 48954.6 | 48875.7 | 22969.2 | 22944.3 |
| BIC | 78531.9 | 78229.4 | 5670.2 | 5702.2 | 49032.7 | 49005.9 | 23041.9 | 23065.4 |
| ICC | 0.7 | 0.7 | 0.6 | 0.6 | 0.7 | 0.6 | 0.7 | 0.7 |
| RMSE | 0.30 | 0.30 | 0.31 | 0.31 | 0.31 | 0.31 | 0.28 | 0.28 |

Table A4. The relationship between the intersection of age, immigration background, and sex and disability. Estimates from random-effects models. Women aged 30-80, SOEP waves 2002-2019, Germany. Models weighted with inverse probability weighting.

|  | Total population | | Primary education | | Secondary education | | Tertiary education | |
| --- | --- | --- | --- | --- | --- | --- | --- | --- |
|  | M1_tot | M2_tot | M1_pr. | M2_pr. | M1_sec. | M2_sec. | M1_ter. | M2_ter. |
| **Intercept** | -1.406 (0.038)*** | -1.251 (0.041)*** | -0.726 (0.115)*** | -0.554 (0.120)*** | -1.317 (0.048)*** | -1.070 (0.050)*** | -1.936 (0.080)*** | -1.749 (0.087)*** |
| **Age components of B-splines** | | | | | | | | |
| BS 1 | 0.510 (0.096)*** | 0.702 (0.096)*** | 1.203 (0.299)*** | 1.186 (0.300)*** | 0.512 (0.119)*** | 0.454 (0.119)*** | 0.303 (0.192) | 0.441 (0.193)* |
| BS 2 | 1.860 (0.072)*** | 1.857 (0.071)*** | 1.215 (0.191)*** | 1.239 (0.193)*** | 1.913 (0.089)*** | 1.861 (0.089)*** | 2.284 (0.160)*** | 2.266 (0.158)*** |
| BS 3 | 3.147 (0.066)*** | 3.035 (0.066)*** | 2.746 (0.165)*** | 2.659 (0.164)*** | 2.916 (0.083)*** | 2.699 (0.081)*** | 3.289 (0.155)*** | 3.201 (0.154)*** |
| **Immigration background (ref. non-immigrant)** | | | | | | | | |
| Immigrant | -0.396 (0.168)* | -0.508 (0.180)** | -0.479 (0.275)+ | -0.408 (0.297) | -0.609 (0.277)* | -1.160 (0.299)*** | 0.232 (0.331) | 0.322 (0.354) |
| **Age components of B-splines x immigration background (ref. BS1, non-immigrant)** | | | | | | | | |
| BS1 x immigrant | 1.548 (0.431)*** | 1.570 (0.428)*** | -0.004 (0.708) | -0.208 (0.705) | 1.405 (0.709)* | 2.762 (0.720)*** | 1.097 (0.870) | 0.919 (0.872) |
| BS2 x immigrant | 0.180 (0.268) | 0.123 (0.265) | 1.124 (0.435)** | 1.079 (0.434)* | 0.668 (0.420) | 0.419 (0.418) | -0.599 (0.613) | -0.677 (0.615) |
| BS3 x immigrant | 1.354 (0.306)*** | 1.232 (0.302)*** | 1.007 (0.448)* | 0.742 (0.444)+ | 1.039 (0.499)* | 1.792 (0.504)*** | 0.270 (0.752) | 0.110 (0.755) |
| **Marital status (ref. unmarried)** | | | | | | | | |
| Married |  | 0.009 (0.023) |  | -0.203 (0.064)** |  | 0.005 (0.029) |  | 0.038 (0.049) |
| **Income (ref. Low)** | | | | | | | | |
| Medium |  | -0.167 (0.021)*** |  | -0.038 (0.058) |  | -0.173 (0.026)*** |  | -0.134 (0.053)* |
| High |  | -0.391 (0.025)*** |  | -0.208 (0.087)* |  | -0.361 (0.031)*** |  | -0.341 (0.058)*** |
| **Immigration background x marital status (ref. non-immigrant, unmarried)** | | | | | | | | |
| Immigrant x married |  | 0.039 (0.083) |  | 0.215 (0.145) |  | -0.021 (0.134) |  | 0.223 (0.164) |
| **Immigration background x income (ref. non-immigrant, low)** | | | | | | | | |
| Immigrant x medium |  | 0.137 (0.069)* |  | -0.154 (0.118) |  | 0.114 (0.111) |  | -0.282 (0.148)+ |
| Immigrant x high |  | 0.047 (0.084) |  | -0.064 (0.160) |  | -0.071 (0.136) |  | -0.295 (0.171)+ |
| SD (Intercept pid) | 1.321 | 1.288 | 1.215 | 1.197 | 1.305 | 1.271 | 1.361 | 1.335 |
| Num.Obs. | 80405 | 80405 | 10418 | 10418 | 49084 | 49084 | 20903 | 20903 |
| R2 Marg. | 0.217 | 0.227 | 0.202 | 0.205 | 0.194 | 0.201 | 0.204 | 0.211 |
| R2 Cond. | 0.715 | 0.709 | 0.678 | 0.673 | 0.702 | 0.694 | 0.721 | 0.716 |
| AIC | 90445.6 | 90130.5 | 11954.9 | 11945.7 | 56376.3 | 56280.4 | 21470.6 | 21421.8 |
| BIC | 90529.2 | 90270.0 | 12020.2 | 12054.5 | 56455.6 | 56412.4 | 21542.1 | 21541.0 |
| ICC | 0.6 | 0.6 | 0.6 | 0.6 | 0.6 | 0.6 | 0.6 | 0.6 |
| RMSE | 0.31 | 0.31 | 0.31 | 0.31 | 0.31 | 0.31 | 0.30 | 0.30 |

Table A5. Missing values by migration background, sex and age.

|  | **Disability** | | **Self-rated health** | | |
| --- | --- | --- | --- | --- | --- |
|  | **Non-immigrants** | **Immigrants** | **Non-immigrants** | | **Immigrants** |
|  | N = 150,181 | N = 13,403 | N = 380,945 | | N = 35,585 |
| Non-missing | 139,549 (92.9%) | 12,600 (94.0%) | 380,433 (99.9%) | | 35,538 (99.9%) |
| Missing | 10,632 (7.1%) | 803 (6.0%) | 512 (0.1%) | | 47 (0.1%) |
| **Sex** |  |  |  | |  |
| Men | 70,290 (46.8%) | 6,215 (46.4%) | 179,571 (47.1%) | | 16,974 (47.7%) |
| Women | 79,891 (53.2%) | 7,188 (53.6%) | 201,374 (52.9%) | | 18,611 (52.3%) |
| **Missing by age group** | |  |  | |  |
| (29,35] | 16,826 (11.2%) | 750 (5.6%) | 47,048 (12.4%) | | 1,795 (5.0%) |
| (35,40] | 17,676 (11.8%) | 1,711 (12.8%) | 46,001 (12.1%) | | 4,090 (11.5%) |
| (40,45] | 19,878 (13.2%) | 2,029 (15.1%) | 49,505 (13.0%) | | 5,137 (14.4%) |
| (45,50] | 19,793 (13.2%) | 1,864 (13.9%) | 48,394 (12.7%) | | 5,342 (15.0%) |
| (50,55] | 17,086 (11.4%) | 1,708 (12.7%) | 42,319 (11.1%) | | 5,004 (14.1%) |
| (55,60] | 14,238 (9.5%) | 1,644 (12.3%) | 37,525 (9.9%) | | 4,794 (13.5%) |
| (60,65] | 13,652 (9.1%) | 1,433 (10.7%) | 34,777 (9.1%) | | 3,937 (11.1%) |
| (65,70] | 12,858 (8.6%) | 1,161 (8.7%) | 31,454 (8.3%) | | 2,891 (8.1%) |
| (70,75] | 10,590 (7.1%) | 695 (5.2%) | 25,827 (6.8%) | | 1,669 (4.7%) |
| (75,80] | 7,584 (5.0%) | 408 (3.0%) | 18,095 (4.8%) |  | 926 (2.6%) |

Figure A1. Self-rated health trajectories by age and immigration background, stratified by sex, from pooled OLS models. Individuals aged 30-80, Germany, SOEP waves 1994-2019. Models weighted with inverse probability weighting.

**
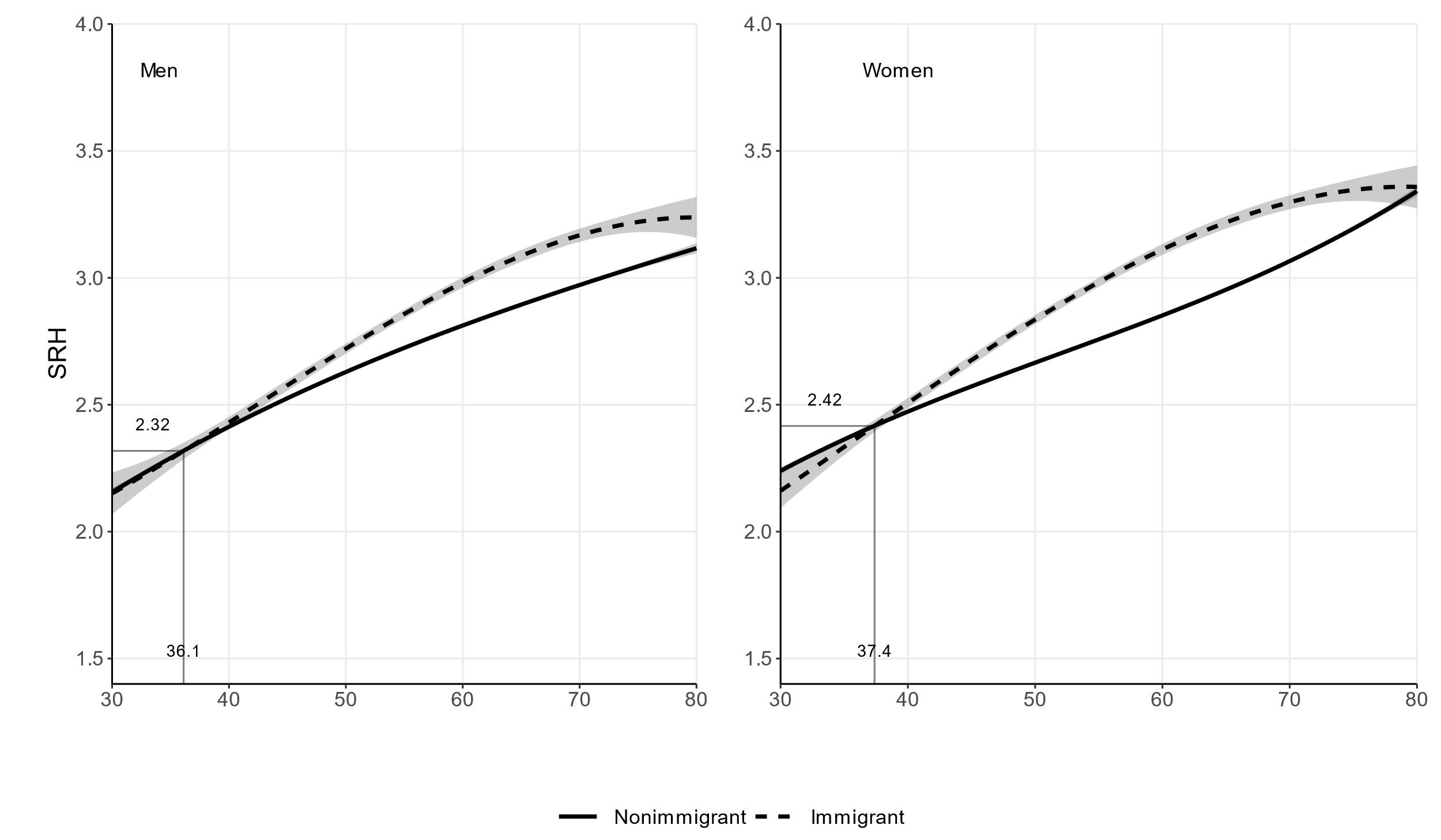
**

Figure A2. Disability trajectories by age and immigration background, stratified by sex, from pooled OLS models. Individuals aged 30-80, Germany, SOEP waves 2002-2019. Models weighted with inverse probability weighting.

**
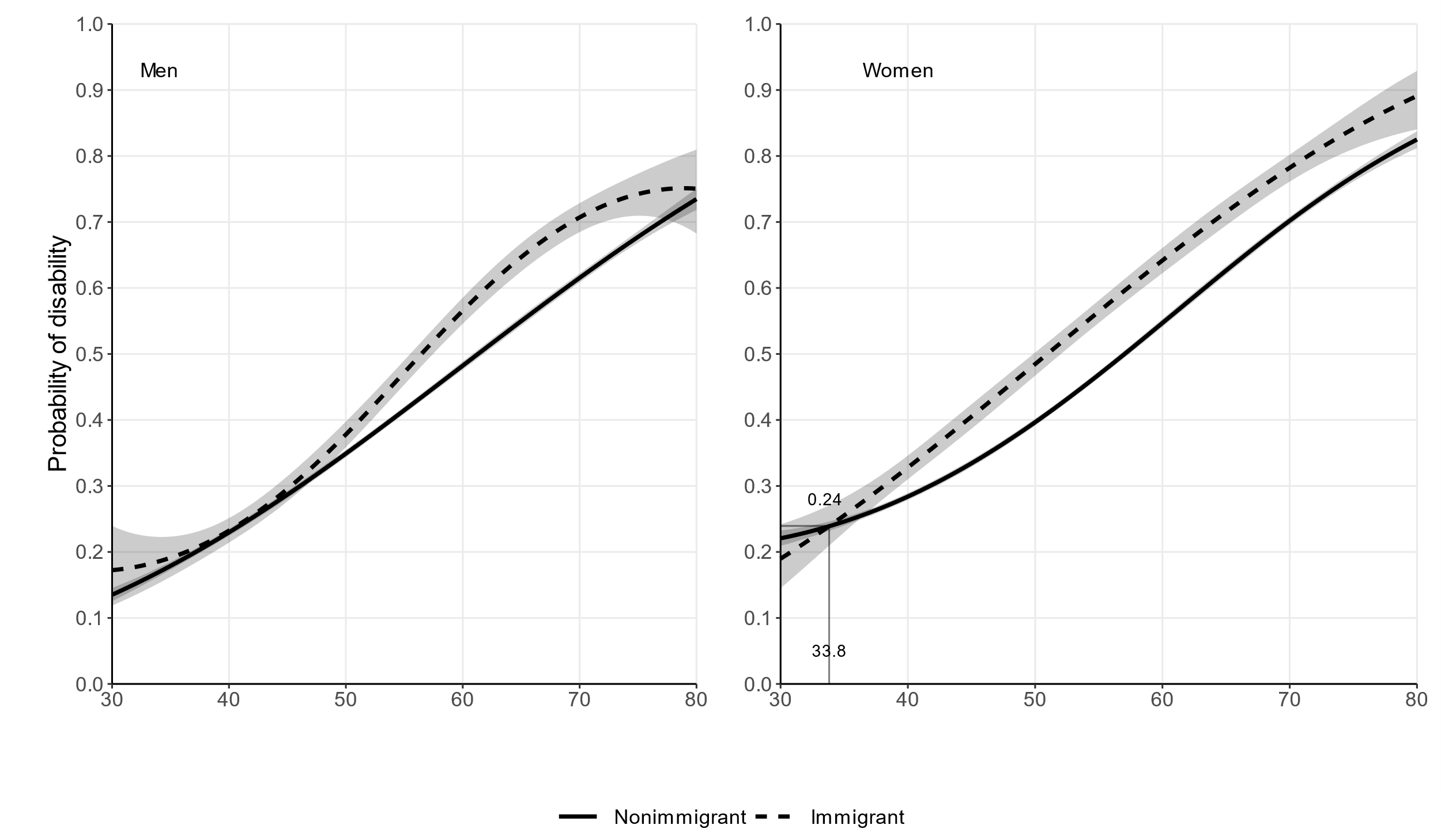
**

Figure A3. Self-rated health trajectories by age and immigration background, stratified by sex, from individual fixed-effects models. Individuals aged 30-80, Germany, SOEP waves 1994-2019. Models weighted with inverse probability weighting.

**
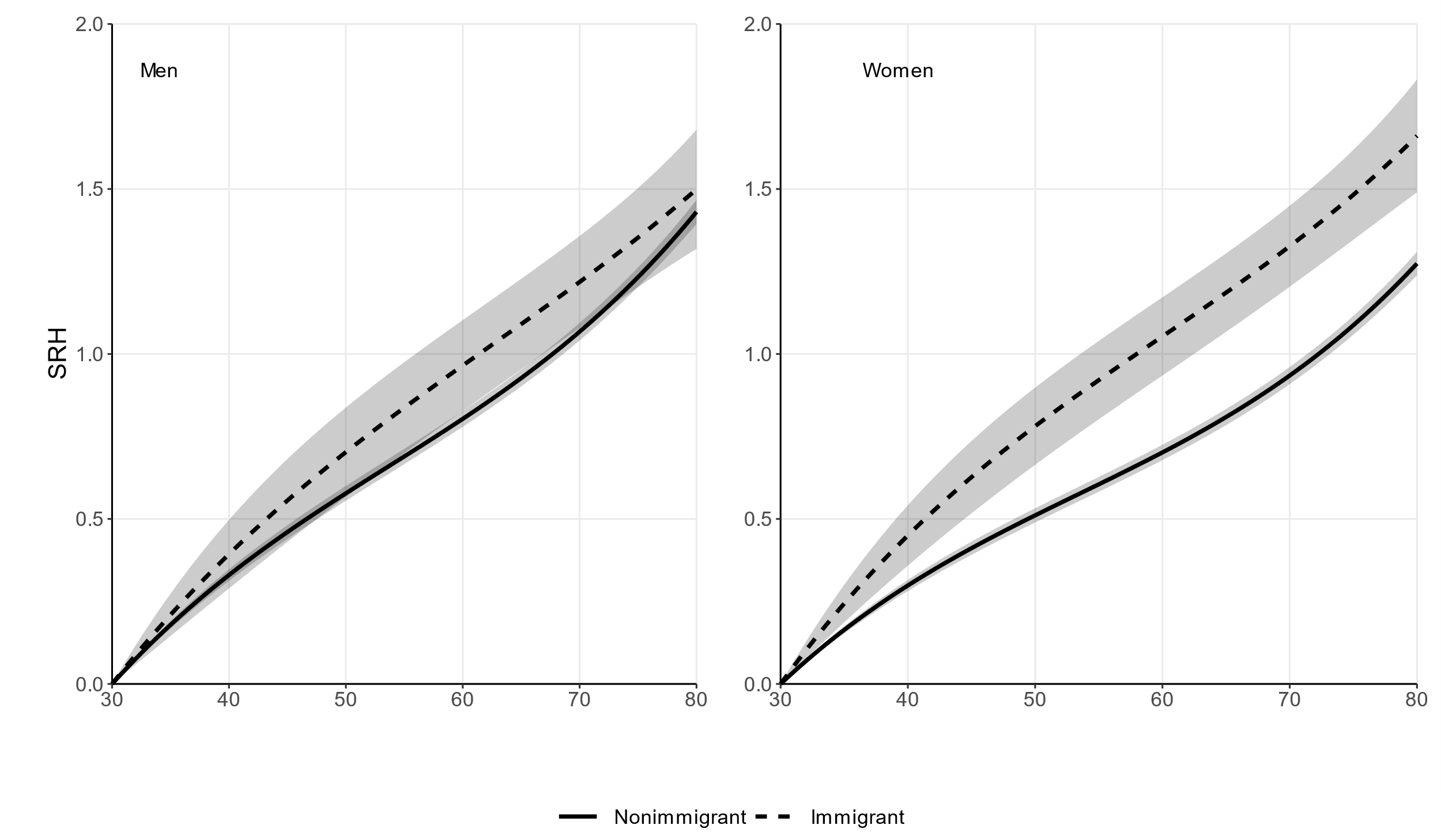
**

Figure A4. Disability trajectories by age and immigration background, stratified by sex, from individual fixed-effects models. Individuals aged 30-80, Germany, SOEP waves 2002-2019. Models weighted with inverse probability weighting.

**
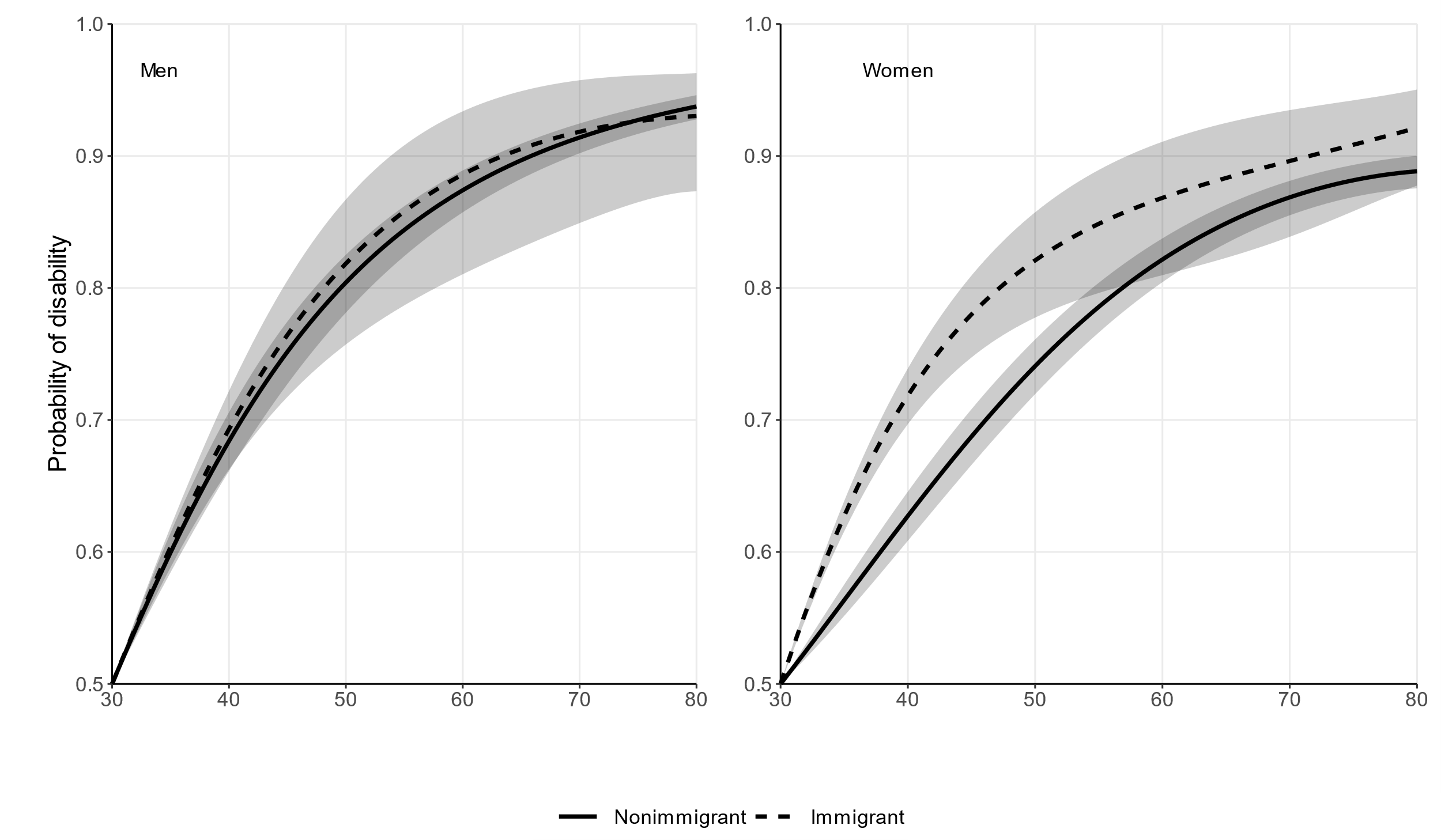
**

Figure A5. Self-rated health trajectories by age and immigration background, stratified by sex and countries of birth. Models adjusted by education and weighted with inverse probability weighting, from random-effects models. Individuals aged 30-80, Germany, SOEP waves 1994-2019.


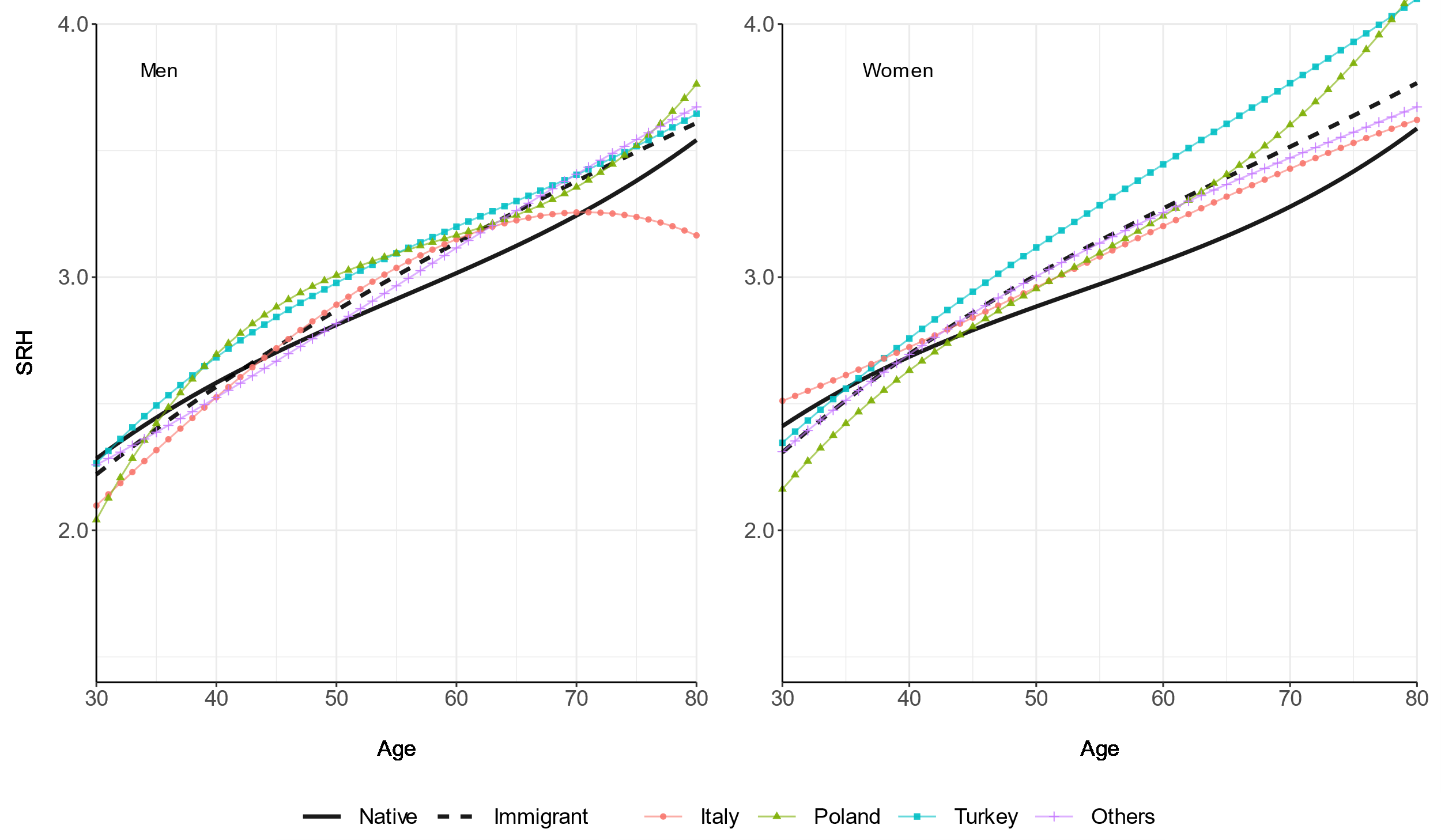


Figure A6. Disability trajectories by age and immigration background, stratified by sex and countries of birth. Models adjusted by education and weighted with inverse probability weighting, from random-effects models. Individuals aged 30-80, Germany, SOEP waves 2002-2019.


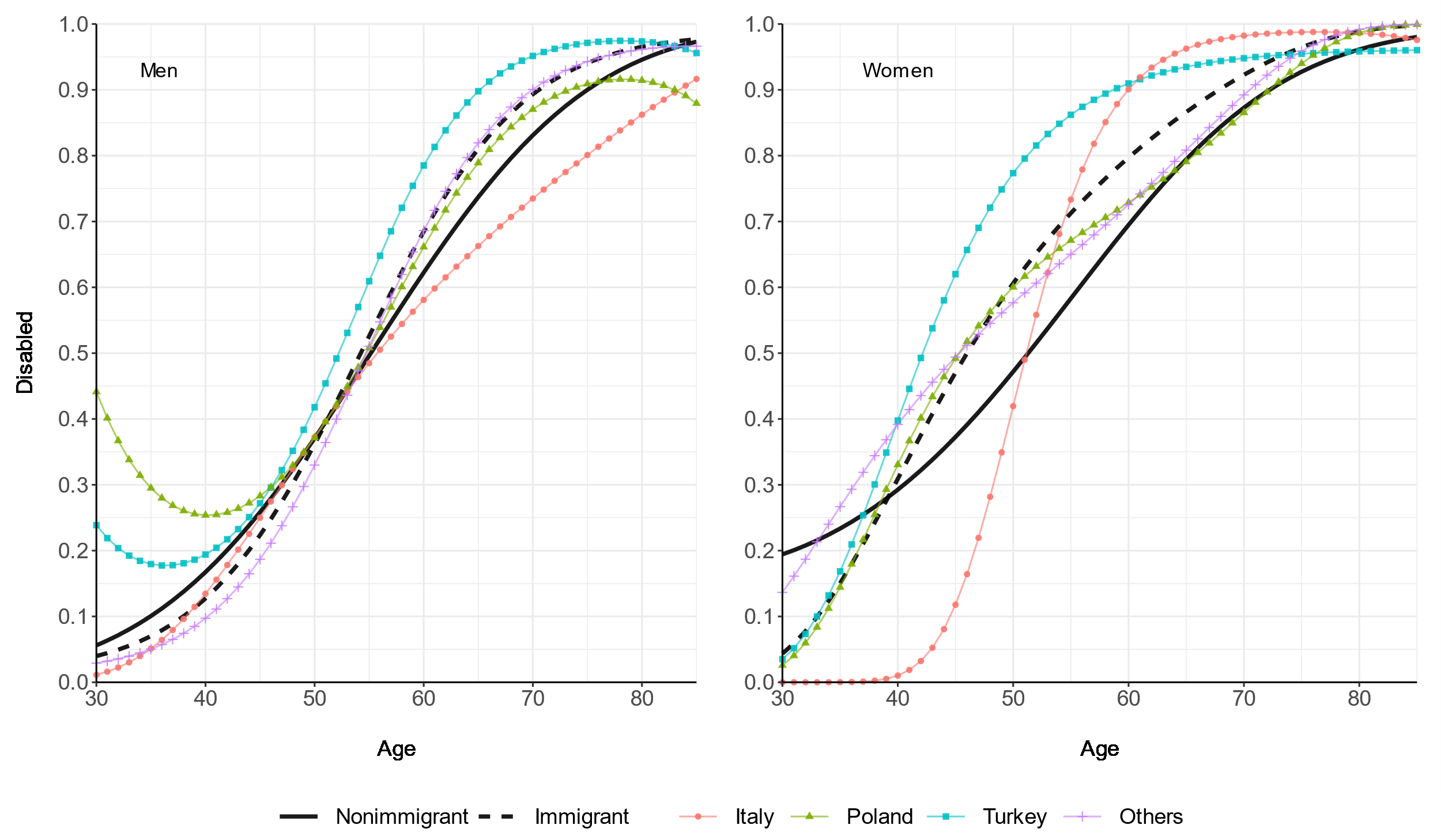


Figure A7. Self-rated health trajectories by age and immigration background, stratified by sex, from random-effects models. Individuals aged 30-80, Germany, SOEP waves 1994-2019. Unweighted Models.


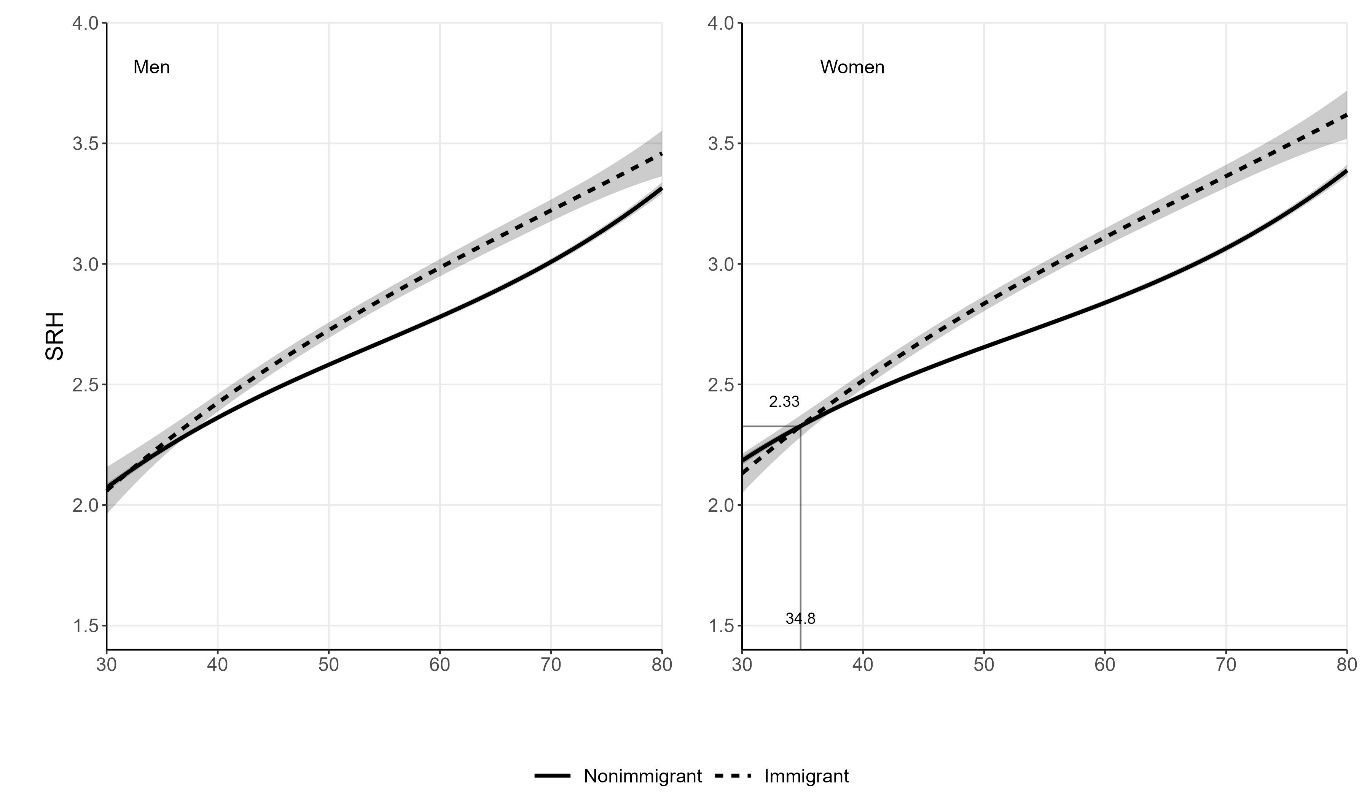


Figure A8. Disability trajectories by age and immigration background, stratified by sex, from random-effects models. Individuals aged 30-80, Germany, SOEP waves 2002-2019. Unweighted Models.

**
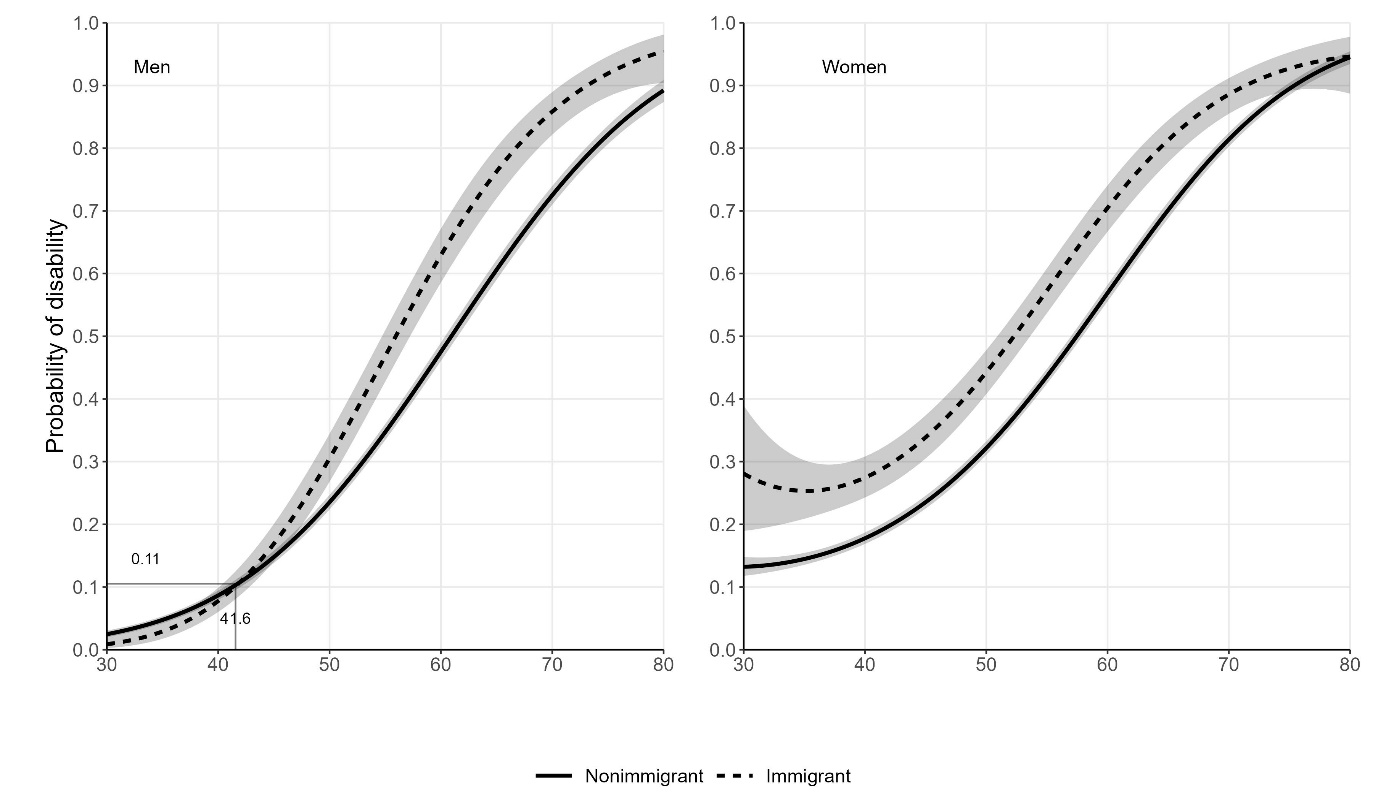
**

Figure A9. Comparison of age profiles estimated via OLS and Ord-logit approaches.

**
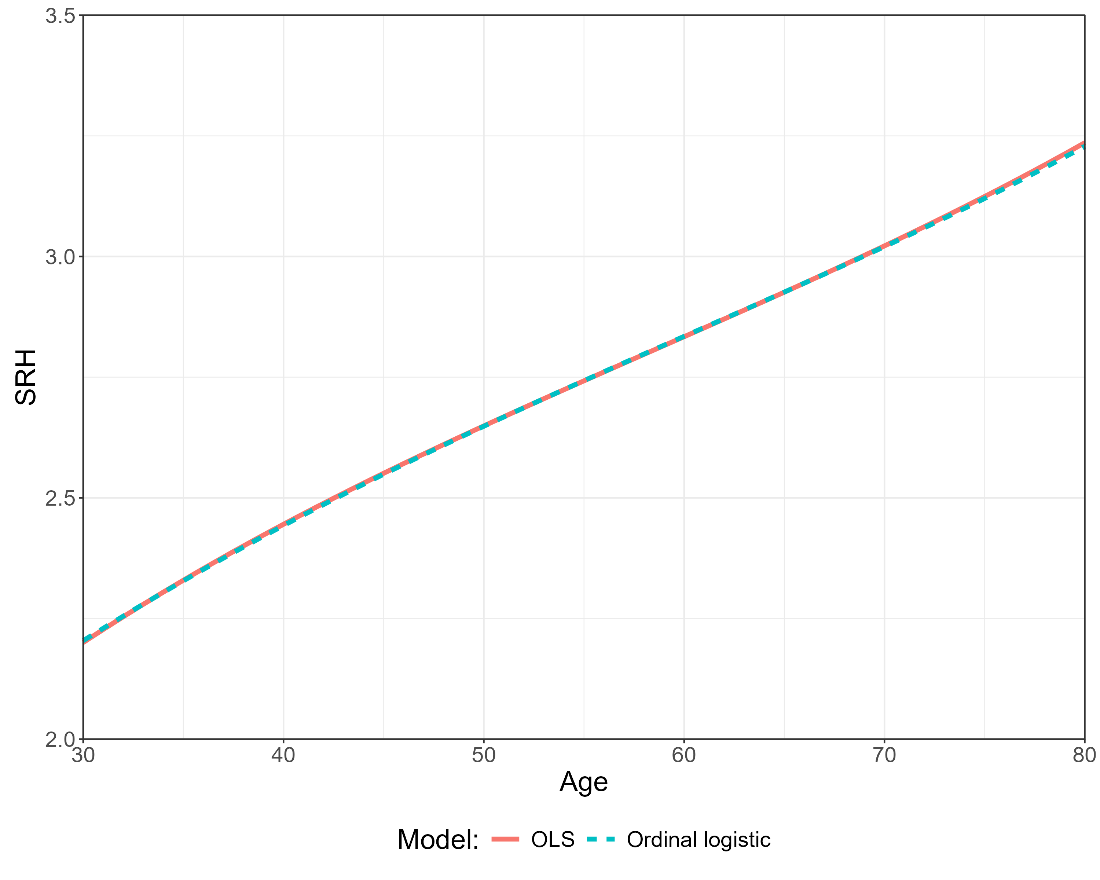
**
